# A Genome-wide Screen Reveals that Reducing Mitochondrial DNA Polymerase Can Promote Elimination of Deleterious Mitochondrial Mutations

**DOI:** 10.1016/j.cub.2019.10.060

**Published:** 2019-12-16

**Authors:** Ason C.-Y. Chiang, Eleanor McCartney, Patrick H. O'Farrell, Hansong Ma

**Affiliations:** 1 Wellcome Trust/Cancer Research UK Gurdon Institute, Tennis Court Road, Cambridge CB2 1QN, UK; 2 Department of Genetics, University of Cambridge, Downing Street, Cambridge CB2 3EH, UK; 3 Department of Biochemistry and Biophysics, University of California, San Francisco, San Francisco, CA 94143, USA

**Keywords:** mito-nuclear interaction, mitochondrial DNA heteroplasmy, mtDNA polymerase gamma, mtDNA competition, mtDNA transmission and inheritance

## Abstract

A mutant mitochondrial genome arising amid the pool of mitochondrial genomes within a cell must compete with existing genomes to survive to the next generation. Even weak selective forces can bias transmission of one genome over another to affect the inheritance of mitochondrial diseases and guide the evolution of mitochondrial DNA (mtDNA). Studies in several systems suggested that purifying selection in the female germline reduces transmission of detrimental mitochondrial mutations [1, 2, 3, 4, 5, 6, 7]. In contrast, some selfish genomes can take over despite a cost to host fitness [8, 9, 10, 11, 12, 13]. Within individuals, the outcome of competition is therefore influenced by multiple selective forces. The nuclear genome, which encodes most proteins within mitochondria, and all external regulators of mitochondrial biogenesis and dynamics can influence the competition between mitochondrial genomes [14, 15, 16, 17, 18], yet little is known about how this works. Previously, we established a *Drosophila* line transmitting two mitochondrial genomes in a stable ratio enforced by purifying selection benefiting one genome and a selfish advantage favoring the other [8]. Here, to find nuclear genes that impact mtDNA competition, we screened heterozygous deletions tiling ∼70% of the euchromatic regions and examined their influence on this ratio. This genome-wide screen detected many nuclear modifiers of this ratio and identified one as the catalytic subunit of mtDNA polymerase gene (*POLG*), *tam*. A reduced dose of *tam* drove elimination of defective mitochondrial genomes. This study suggests that our approach will uncover targets for interventions that would block propagation of pathogenic mitochondrial mutations.

## Highlights

• Multiple nuclear factors affect selective transmission of mitochondrial genomes
• Reducing mtDNA polymerase restricts the transmission of detrimental mtDNA mutants

## Graphical Abstract

**Figure:**
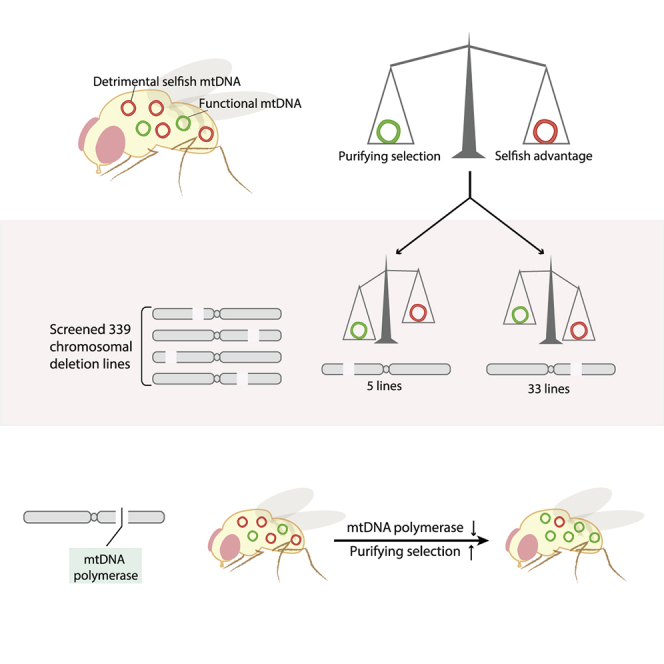

## Teaser

Mitochondrial genomes within an organism compete for transmission. Chiang et al. reveal that multiple nuclear genes can affect this competition. In particular, reducing the nuclear-encoded mtDNA polymerase limits the transmission of detrimental mtDNA mutants. Thus, the nuclear genome can affect mitochondrial disease progression and mtDNA evolution.

## Results and Discussion

**Figure 1:**
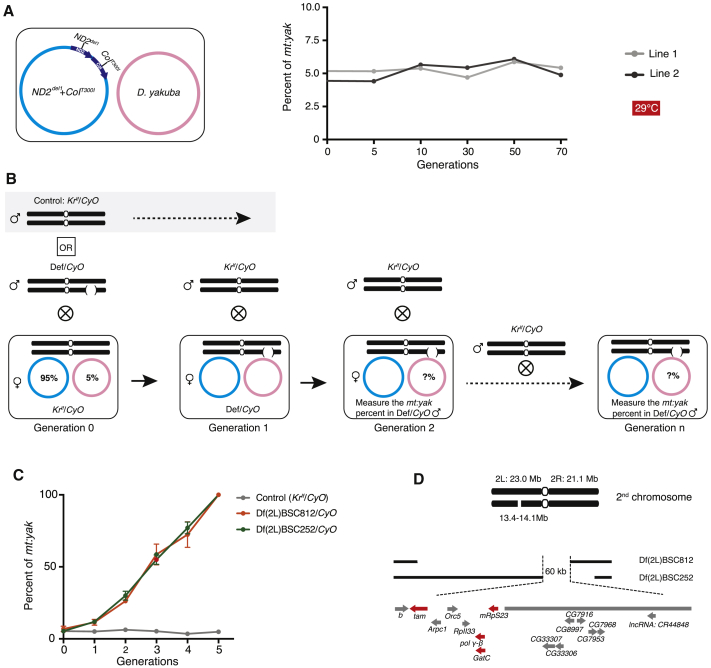
A Deficiency Screen Identified Two Overlapping Deletion Lines that Significantly Increased the Proportion of *mt:yak* (A) The stable heteroplasmic line. The *D. melanogaster* mtDNA (blue circle) has two mutations, *ND2^del1^* and *CoI*^*T300I*^. *ND2*^*del1*^ is a 9-bp in-frame deletion in the gene encoding NADH-dehydrogenase 2 (dark blue); it is a viable hypomorphic allele. *CoI*^*T300I*^ is a temperature-sensitive allele of cytochrome *c* oxidase I (dark blue); when homoplasmic, it is lethal at 29°C but viable at lower temperatures. Although *mt:yak* (pink circle) is fully functional in *D. melanogaster*, it is usually outcompeted by the endogenous wild-type *D. melanogaster* mtDNA. However, when paired with *mt:ND2*^*del1*^+*CoI*^*T300I,*^*mt:yak* is stably transmitted at ∼5% for over 70 generations at 29°C [8]. (B) The genetic cross scheme to introduce deletion chromosomes (Table S1) into the stable heteroplasmic line (only shown for the 2^nd^ chromosome deficiencies schematized by a bracketed interruption). First, 10 *Kr*^*If*^/*CyO* heteroplasmic females (generation 0) were crossed to 5 deficiency/*CyO* (Def/*CyO*) males to generate 10–20 female progeny with the genotype of Def/*CyO* (generation 1), which were further crossed to 10 *Kr*^*If*^/*CyO* males to produce progeny (generation 2). Def/*CyO* males of generation 2 were collected to measure the *mt:yak* percentage via qPCR. For some deficiencies, Def/*CyO* males were collected at multiple generations to follow the *mt:yak* percentage over time. In controls, *Kr*^*If*^/*CyO* males were used for the first cross instead of Def/*CyO* males. All the subsequent steps were the same, and *Kr*^*If*^/*CyO* males of generation 2 were collected to measure the *mt:yak* percentage. (C) Two overlapping deficiencies (balanced by *CyO*) increased *mt:yak* transgenerationally (see also Tables S2 and S3). The *mt:yak* percentage reached 100% after five generations. Error bars indicate SDs of three independent experiments. (D) The region deleted in both deficiencies contains 15 genes, four of which encode mitochondrial proteins (red) (also refer to Figure S1).

*Drosophila melanogaster* tolerates heterozygous deletions well, and collections of chromosomal deletions (i.e., deficiencies) tiling 98% of the euchromatic genome have been developed for genetic screening purposes [19, 20]. To uncover nuclear regions that regulate mitochondrial DNA (mtDNA) competition, we performed a deficiency screen on a heteroplasmic line that stably transmits two genomes: a functional mtDNA from *Drosophila yakuba* (*mt:yak*) and a temperature-sensitive lethal genome harboring two mutations, *mt:ND2*^*del1*^+*CoI*^*T300I*^ from *D. melanogaster* (Figure 1A) [8]. Previously, we showed that the *D. melanogaster* genome has a selfish transmission advantage, whereas *mt:yak* is favored by purifying selection because it provides the functional *mt:CoI*. The two opposing selections are balanced so that the heteroplasmic ratio (∼5% *mt:yak* to ∼95% *mt:ND2*^*del1*^+*CoI*^*T300I*^) was stable for over 70 generations at the restrictive temperature (29°C) of the *mt:ND2*^*del1*^+*CoI*^*T300I*^ genome (Figure 1A). We reasoned that a balance of two selective forces would be very sensitive to perturbation. Perhaps just a change in the gene dose of nuclear genes modulating competition would alter the heteroplasmic ratio.

For the screen, 339 deletion chromosomes covering most of chromosomes II and III were introduced into the heteroplasmic flies (Table S1). Sixty-three crosses produced no or few progeny carrying the deletion (Table S1). Although it is likely that the lethality for some of the crosses was due to an inability to maintain the functional *mt:yak* genome, here we focus only on the 276 lines that produced progeny; in these, we measured the *mt:yak* percentage in adult males one generation after the deletion was introduced (i.e., generation 2; Figure 1B). Five lines had a substantially higher percentage of *mt:yak* (≥ 10%), whereas 33 lines had a lower percentage (≤ 2%) (Tables S2 and S3). More than 10% of the tested lines changed the *mt:yak* percentage, leading us to conclude that multiple nuclear factors directly or indirectly regulate competition between mitochondrial genomes.

Of the 38 deficiencies that altered the *mt:yak* percentage, the two (BSC252 and BSC812) causing the largest increase (from ∼5% to ∼28%) partially overlap (Table S3; Figures 1C and 1D). This suggests that a locus lying in the 60 kb region of the 2^nd^ chromosome removed by both deletions is responsible for the observed phenotype (Figure 1D). Consistent with a common defect, both deletions caused a progressive and parallel rise in *mt:yak* over multiple generations until it reached 100% at generation 5 (Figure 1C). To produce this progressive heritable rise, these deletions must give *mt:yak* a selective advantage in the germline allowing accumulation in each generation. To confirm and further narrow down the responsible region, we tested two more deletions: Exel7059 (lacking the entire 60 kb region) and FDD-0428643 (only lacking the left 15 kb segment of the 60 kb region). Both gave the same increase in the *mt:yak* percentage (Figure S1A). This confined our candidates to eight genes, including four with known mitochondrial functions: mtDNA polymerase catalytic subunit polymerase gene *POLG* (*tam*), mtDNA polymerase accessory subunit *POLG2* (*pol* γ-β), glutamyl tRNA-aminotransferase *GatC*, and mitochondrial ribosomal protein *mRpS23* (Figure S1B).

**Figure 2:**
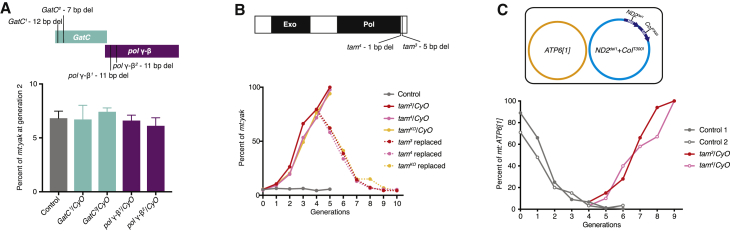
Reducing *tam* Gene Dose Increased the Abundance of Functional mtDNA in Two Heteroplasmic Lines (A) Reducing the dose of *pol* γ-β or *GatC* showed no effect on mtDNA competition. Top: a schematic shows the genomic arrangement of the co-transcribed genes *GatC* and *pol* γ-β with the position and description of loss-of-function mutants isolated via CRISPR/Cas9-based editing (Figure S2). Bottom: the histogram shows the *mt:yak* percentage for different nuclear genotypes. Error bars indicate SDs of three independent experiments. (B) Heterozygosity for any of the three mutant alleles of *tam* over the *CyO* chromosome dramatically shifted the heteroplasmic ratio (see Figure S3 for the total mtDNA copy number of the *tam* heterozygotes). Top: a schematic of Tam protein shows functional domains and the positions of two homozygous lethal mutations, *tam*^*3*^ and *tam*^*4*^. At 29°C, the *mt:yak* percentage increased in *tam*^*3*^, *tam*^*4*^, and *tam*^*KO*^ heterozygous mutants with a speed similar to that observed with the BSC252 and BSC812 deficiencies (Figure 1C). Chromosomes bearing *tam* mutations were introduced at generation 0 and the *mt:yak* percentage was followed over generations via qPCR (solid line). From generation 1 to 3, only progeny heterozygous for the *tam* mutation were followed and crossed to *Kr*^*If*^/*CyO* to examine the cross-generational dose effect of *tam*. At generation 4, the female progeny lacking the *tam* mutation were also mated with *Kr*^*If*^/*CyO*, and these populations were maintained in parallel for another 6 generations to assess the *mt:yak* percentage (dotted lines). (C) Reducing *tam* dose also increased the percentage of the functional genome (*mt:ATP6[1]*) in the *mt:ATP6[1]*/*mt:ND2*^*del1*^+*CoI*^*T300I*^ line. The percentage of *mt:ATP6[1]* in two heteroplasmic lineages (control 1 and control 2) was measured over generations at 29°C. At generation 4, female progeny were divided into two populations: one was mated with males heterozygous for *tam*^*3*^ or *tam*^*4*^ to remove one functional copy of *tam* and maintained in the heterozygous *tam* mutant background (balanced by *CyO*) for the subsequent generations; the other population was mated with *Kr*^*If*^/*CyO* males as controls. All the controls refer to *Kr*^*If*^/*CyO*.

To investigate whether *pol* γ-β and its neighboring and co-transcribed gene *GatC* play a role in regulating mtDNA transmission, we generated loss-of-function mutants via CRISPR/Cas9-based editing (Figure S2). Heterozygous mutants of *pol* γ-β and *GatC* did not alter *mt:yak* percentages, suggesting neither was responsible for the changes in the heteroplasmic ratio (Figure 2A). We then tested three *tam* mutants: two classical mutations, *tam*^*3*^ and *tam*^*4*^, which contain small deletions that shift the reading frame [21], and *tam*^*KO*^, which removes the entire coding region including the UTRs [22]. Heterozygosity for each of these *tam* mutations increased the *mt:yak* percentage just as observed with the four deficiencies (Figure 2B). By generation 5, *mt:yak* took over. To test the reversibility of the effect, we restored a wild-type *tam* genotype in some flies having a residual ∼20% of *mt:ND2*^*del1*^+*CoI*^*T300I*^ at generation 4 and followed over subsequent generations (Figure 2B). In these flies, selection reversed its course and the *mt:yak* percentage declined. After four generations, *mt:yak* levels re-balanced at ∼5%. Of note, in accord with the gene dose, the mRNA level of *tam* is halved in the deletion lines (Figure S3A). Despite the dramatic shifts in the relative abundance of the two genomes, only minor fluctuations in total mtDNA copy number were detected in the newly laid eggs and adult flies (Figure S3B; also shown in [22]). We conclude that removing one functional genomic copy of *tam* but not *pol* γ-β or *GatC* increased the *mt:yak* percentage in the given nuclear background.

We were somewhat surprised that the dose of the gene encoding the catalytic subunit of POLG modified competition whereas the dose of the essential accessory subunit did not. To pursue this further, we tested other genes involved in mtDNA replication (*twk*, *mtSSB*, *TFAM*, and *Top3*α) and did not detect modification of the heteroplasmic ratio in a manner dependent on the dose of these genes (Figure S3C). Thus, at least in the context of our screen, the input of *tam* appears to be relatively specific among replication functions.

To test whether the influence of *tam* dose on mtDNA competition extended beyond the specific mtDNA pair used in the screen, we examined a different pairing of mitochondrial genomes. We previously showed that when the *mt:ND2*^*del1*^+*CoI*^*T300I*^ genome is paired with a distantly related *D. melanogaster* mitochondrial genome, *mt:ATP6[1]*, the temperature-sensitive mutant genome exhibits such a powerful selfish advantage that it overrides the constraint of purifying selection; the mutant genome replaced the complementing *mt:ATP6[1]* over a few generations, leading to the death of the entire lineage at 29°C [23]. We re-established two independent lineages of this unstable heteroplasmy, and *mt:ND2*^*del1*^+*CoI*^*T300I*^ percentages rose rapidly as expected (Figure 2C). Before the demise of the stock, we removed one copy of functional *tam* by introducing chromosomes bearing the *tam*^*3*^ or *tam*^*4*^ mutation in flies at generation 4, which still had ∼10% *mt:ATP6[1]*. Instead of observing a continued decline of *mt:ATP6[1]* as in control flies, *mt:ATP6[1]* percentages increased over successive generations in the two lines with introduced *tam* mutations (Figure 2C). After five generations, *mt:ATP6[1]* reached 100%. We conclude that reducing the gene dose of *tam* can increase the transmission of a distinct functional mitochondrial genome.

**Figure 3:**
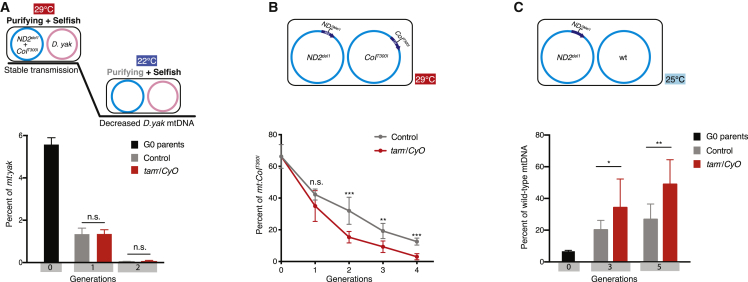
Reducing *tam* Gene Dose Enhances Purifying Selection (A) *tam* heterozygosity showed no effect on the dynamics of the *mt:yak* decline when the *mt:yak*/*mt:ND2*^*del1*^+*CoI*^*T300I*^ line was cultivated at 22°C, where the purifying selection against the *mt:ND2*^*del1*^+*CoI*^*T300I*^ genome was significantly reduced. In both control and *tam* mutant backgrounds (*tam*^*3*^, *tam*^*4*^, or BSC252; balanced by *CyO*), *mt:yak* was eliminated in two generations. (B) The decline of *mt:CoI*^*T300I*^ in the *mt:ND2*^*del1*^/*mt:CoI*^*T300I*^ line at the restrictive temperature was accelerated in heterozygous *tam* mutants. The percentage of *mt:CoI*^*T300I*^ was followed in various flies with only one functional copy of *tam* (*tam*^*3*^, *tam*^*KO*^, BSC252, or BSC812; balanced by *CyO*) and *Kr*^*If*^/*CyO* nuclear background over generations. (C) Reducing *tam* gene dose enhanced the rate at which wild-type mtDNA overtook *mt:ND2*^*del1*^ at 25°C. The percentage of the wild-type mtDNA was followed in various heterozygous *tam* mutants (*tam*^*3*^, *tam*^*KO*^, BSC252, or BSC812; balanced by *CyO*) and *Kr*^*If*^/*CyO* nuclear background. Error bars indicate SDs of three or more independent experiments; p values: Student’s t test. All the controls refer to *Kr*^*If*^/*CyO*.

In the two tested heteroplasmic lines, mtDNA competition is influenced by both purifying and selfish selection, so the effect of *tam* dose could be due to enhancement of purifying selection to benefit the functional genome (*mt:yak* or *mt:ATP6[1]*) or diminution of the selfish transmission advantage of *mt:ND2*^*del1*^+*CoI*^*T300I*^, or both. To address this, we transferred the *mt:yak*/*mt:ND2*^*del1*^+*CoI*^*T300I*^ lines to a lower temperature (22°C), where purifying selection against the *mt:ND2*^*del1*^+*CoI*^*T300I*^ is greatly diminished because of improved function of the mutant [5]. At this temperature, selfish selection dominates the competition, and *mt:yak* percentage declined and became undetectable in two generations in control fly groups (Figure 3A; also described in [8]). When *tam* was heterozygous, *mt:yak* declined just as it did in control flies (Figure 3A). Thus, at least in this experimental context, the dose of *tam* has little or no effect when selfish selection has the dominant influence. Either *tam* dose does not act on selfish selection or *tam* dose is unable to act on selfish selection at this temperature.

Next, we examined the effect of *tam* dose in a heteroplasmic combination where only purifying selection affects mtDNA competition. We previously showed that when the *mt:ND2*^*del1*^ genome was paired with the *mt:CoI*^*T300I*^ genome, *mt:ND2*^*del1*^ steadily outcompetes *mt:CoI*^*T300I*^ because of a difference in the oxidative phosphorylation (OXPHOS) function at 29°C [5]. These two genomes share the same non-coding region, and differ only by mutations in *mt:ND2* and *mt:CoI*, so there is no selfish selection involved. We found that in flies where *tam* dose was reduced, the decline of *mt:CoI*^*T300I*^ was accelerated (Figure 3B), suggesting that reducing *tam* enhances purifying selection. To further test whether *tam* dose acts only on *mt:CoI*^*T300I*^ or is only manifested at 29°C, we generated another heteroplasmic line where the *mt:ND2*^*del1*^ mutant was paired with the wild-type mtDNA and followed the heteroplasmy dynamics at 25°C. The *mt:ND2*^*del1*^ allele is slightly compromised for OXPHOS function and is slowly displaced by the wild type because of a weak purifying selection [5] (Figure 3C). In lines with only one functional genomic copy of *tam*, the wild-type mtDNA took over faster (Figure 3C). Thus, reducing *tam* acts to enhance two distinct examples of purifying selection.

**Figure 4:**
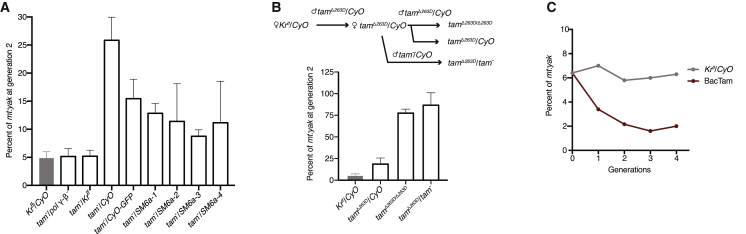
Modulating Tam Function Alone Is Sufficient to Influence Competition between Mitochondrial Genomes (A) The dose effect of *tam* on the percentage of *mt:yak* was not detected in nuclear backgrounds where 2^nd^ chromosomes unrelated to the *CyO* balancer were used. The *mt:yak* percentage was followed in various heterozygous *tam* mutants (*tam*^*3*^, *tam*^*KO*^, BSC252, or BSC812) balanced by *CyO*-related or unrelated 2^nd^ chromosomes. A detailed cross scheme for each genotype is presented in Figure S4D. Error bars indicate SDs of three or more independent experiments. (B) Homozygous or transheterozygous viable *tam* mutants showed that reducing functional Tam alone is sufficient to increase the percentage of *mt:yak* (see also Figure S4). Top: the cross scheme used to introduce various *tam* alleles into the stable heteroplasmic line. The *mt:yak* percentage was followed in *tam*^Δ*263D*^/*CyO*, *tam*^Δ*263D*^/*tam*^Δ*263D*^, and *tam*^Δ*263D*^/*tam*^*−*^ (*tam*^*3*^*or tam*^*KO*^) adult males (see Figure S2 for the sequence details of *tam*^Δ*263D*^). Error bars indicate SDs of three or more independent experiments. (C) Increasing *tam* dose decreased the percentage of *mt:yak*. The stable heteroplasmic females were crossed to males homozygous for *tam-GFP* on the 3^rd^ chromosome (BAC clone; a gift from Hong Xu, NIH) for two generations to produce heteroplasmic flies containing two endogenous copies of *tam* on the 2^nd^ chromosome and two extra copies of *tam-GFP* on the 3^rd^ chromosome. The *mt:yak* percentage was followed for four generations.

For all the experiments described above, genetic crosses were designed to minimize nuclear background differences and all tested deletions and mutations were heterozygous with a balancer chromosome (*CyO* for all presented data and *TM6B* for the 3^rd^ chromosome deficiencies; see Figure 1B and STAR Methods). However, this left open the possibility that the dose effect on heteroplasmy dynamics we observed for *tam* is specific for the given nuclear background. To test whether this is the case, we altered the crossing scheme and examined the consequence of *tam* heterozygosity with different second chromosomes. We found that, when chromosomes unrelated to the *CyO* balancer were used, the dose of *tam* had only negligible effect on the *mt:yak* percentage (Figure 4A). This suggests that the *CyO* chromosome carries one or more polymorphisms that synergize with the *tam* dose to create the observed phenotype. We hypothesized that the *CyO* balancer provided less *tam* function than other chromosomes (hypomorphic for *tam*). Such behavior might be attributed to either direct changes in the *tam* sequence or other modifying mutations that reduce the functional output of Tam. We tested various balancer chromosomes with related origins but different polymorphisms in the *tam* sequence. Although heterozygosity for *tam* gave a phenotype with all of these balancers, the strength of the phenotype varied. This variation combined with sequences of *tam* from these balancers (Figure S4A) and a test of *tam* expression from *CyO* (Figure S4B) suggests that the modification is complex and might either be caused by diverse polymorphisms associated with *tam* on the balancer or unrelated modifiers.

To avoid the complicated genetic interactions with the *CyO* balancer, we tested whether a more substantial change in *tam* alone is sufficient to modulate mtDNA competition. We used CRISPR/Cas9-based mutagenesis to isolate mutations missing a single amino acid in the highly conserved exonuclease domain of *tam* (*tam*^Δ*262Y*^ and *tam*^Δ*263D*^) (Figure S2). These alleles are homozygous viable, and also transheterozygous viable with *tam*^*3*^, *tam*^*4*^, and *tam*^*KO*^. We found that after introducing two copies of *tam*^Δ*262Y*^ or *tam*^Δ*263D*^ in the *mt:yak/mt:ND2*^*del1*^+*CoI*^*T300I*^ line, the *mt:yak* percentage increased to over 75% at generation 2. Similarly, the *mt:yak* percentage in transheterozygous *tam*^*−*^/*tam*^Δ*262Y*^ or *tam*^*−*^/*tam*^Δ*263D*^ increased to over 70% at generation 2 (Figures 4B and S4C). These data suggest that compromising *tam* function alone is sufficient to modify the competition between mitochondrial genomes, but Tam function probably needs to be reduced to less than 50%. Additionally, we analyzed the consequence of an increase in *tam* dose by following flies with two extra copies of *tam* (BacTam). Over a few generations at 29°C, the percentage of *mt:yak* fell from 6% to 2% in the BacTam background (Figure 4C). All the above data suggest that altering *tam* functional output alone is sufficient to influence the transmission of detrimental mitochondrial genomes.

Our findings show that the balance that maintains a stable heteroplasmic state is precarious and modified by many genetic loci and that the shift in function of one of these, *tam*, can drive the elimination of a detrimental mitochondrial mutation that was otherwise stably inherited for many generations. The discovery that the gene involved encodes the mtDNA polymerase suggests a connection with replication of the genomes, but the genetic analysis reported here does not directly reveal the mechanism. It is tempting to speculate that reducing Tam activity might favor the replication of the diverged *mt:yak* genome, but no such favoritism was observed when the temperature was reduced to minimize the functional difference between *mt:yak* and the *mt:ND2*^*del1*^+*CoI*^*T300I*^ genome. Furthermore, at 25°C, the lower dose of *tam* still favored the functional genome when two *D. melanogaster* mitochondrial genomes were pitted against each other (Figure 3C). This suggests that the differential effect of *tam* dose impacts the effectiveness of purifying selection. Although speculative, such an effect might be explained by the involvement of Tam in a newly advanced quality control mechanism. Recently, Zhang et al. showed that PINK1, a kinase sensitive to mitochondrial potential, is selectively stabilized on the surface of mitochondria enriched for mutant genomes [24]. They further showed that PINK1 phosphorylates Larp to inhibit local translation of nuclear-encoded mitochondrial proteins on the surface of the unfit mitochondria. Tam was one of the factors whose expression was dramatically reduced by this signaling pathway. They proposed that reduced translation starves unfit mitochondria of nuclear-encoded replication factors. Accordingly, Tam could be a key factor limiting replication in unfit mitochondria when it falls below a certain threshold. A reduction in the dose could promote the action of this system by making it easier to reach the threshold that starves the unfit mitochondria of this limiting factor. However, cell biological and disease phenotypes of *POLG* mutations are diverse, suggesting the existence of alternative possible explanations for how dose change can impact the balance of heteroplasmic genomes [25, 26, 27, 28].

Regardless of the mechanism, our genetic findings reveal numerous nuclear loci that affect the competition between mitochondrial genomes, suggesting that multiple pathways influence the selective forces defining the outcome of competition. Perhaps reflecting complex inputs, the magnitude of the impact of *tam* gene dose differs strikingly in different genetic backgrounds. In the two examples where diverged mitochondrial genomes are differently favored by selfish and purifying selection, reduction of *tam* dose completely reverses the outcome of the competition such that the winner becomes the loser and is eliminated (Figures 2B and 2C). This outcome seems out of proportion with subtler shifts in the strength of purifying selection assessed in other heteroplasmic backgrounds (Figures 3B and 3C). Perhaps as-yet unappreciated differences in the interaction of Tam with the much altered regulatory regions of these competing genomes increase the sensitivity to *tam* dose in these competitions.

In conclusion, the genetic approach we have used here has the potential of defining in some detail the largely unknown rules of nuclear management of mtDNA transmission (e.g., [14, 15, 16, 17, 18]). The pervasive impact of a change in the level of mtDNA polymerase catalytic subunit shows the potency of the nuclear influence on the success of mitochondrial genomes, a factor that would affect the heritance of heteroplasmic mitochondrial disease traits. The large number of loci that influence selection suggests that nuclear management of mitochondrial evolution is deeply entrenched. We propose that it has played a role throughout eukaryotic evolution in taming and subjugating the genome of an infecting microbe to adopt its current role. Because many mitochondrial diseases are carried in heteroplasmy, the extensive nuclear inputs might identify pharmacologically accessible pathways whose manipulation could provide clinical benefit.

## STAR★Methods

### Key Resources Table

**Table:** 

REAGENT or RESOURCE	SOURCE	IDENTIFIER
**Experimental Models: Organisms/Strains**		

*D. melanogaster*: Bloomington Deficiency Kit	Bloomington *Drosophila* Stock Center	https://bdsc.indiana.edu/stocks/df/dfkit.html
*D. melanogaster*: *twk* mutants (*twk*^*1*^ and *twk*^*2*^), *GatC* mutants (*GatC*^*1*^ and *GatC*^*2*^), *tam* mutants (*tam*^Δ*262Y*^ and *tam*^Δ*263D*^), *pol γ-β* mutants (*pol* γ-β^*1*^ and *pol* γ-β^*2*^), see Figure S2	This paper	N/A
*D. melanogaster: tam*^*KO*^	[22]	N/A
*D. melanogaster:* BacTam	Hong Xu (NIH)	N/A
*D. melanogaster*: *tam*^*3*^	Bloomington *Drosophila* Stock Center	BDSC:3410
*D. melanogaster*: *tam*^*4*^	Bloomington *Drosophila* Stock Center	BDSC:25145
*D. melanogaster*: Df(2L)Exel7059,	Bloomington *Drosophila* Stock Center	BDSC:7826
*D. melanogaster*: Df(2L)FDD-0428643	Bloomington *Drosophila* Stock Center	BDSC:25166
*D. melanogaster*: Df(2L)Exel7043	Bloomington *Drosophila* Stock Center	BDSC:7816
*D. melanogaster*: *nos-Cas9*	Bloomington *Drosophila* Stock Center	BDSC:54591

**Oligonucleotides**		

Primers for qPCR, see Table S4	This paper	N/A
Primers for RT-qPCR, see Table S4	This paper	N/A

**Recombinant DNA**		

pCDF5+guide RNA for CRISPR/Cas9-based editing for the following genes (*twk*, *GatC*, *tam* and *pol* γ-β)	This paper	N/A

**Software and Algorithms**		

Guide RNA design for the following genes (*twk*, *GatC*, *tam* and *pol* γ-β)	FLYCRISPR	https://flycrispr.org/

**Other**		

Sequence data of *tam* ROF of a number of 2^nd^ chromosomes (*D. melanogaster*), See Figure S4A	This paper	N/A

### Lead Contact and Materials Availability

Further information and requests for resources and reagents should be directed to and will be fulfilled by the Lead Contact, Hansong Ma (hm555@cam.ac.uk). There are no restrictions to the availability of reagents.

### Experimental Model and Subject Details

The following flies were used in this study: Bloomington Deficiency Kits (Table S1), additional deficiency lines that cover the entire *tam* gene (Df(2L)Exel7059 and Df(2L)FDD-0428643) or *twk* gene (Df(2L)Exel7043) (BDSC:7826, BDSC:21566 and BDSC:7816, respectively), *tam*^*3*^ and *tam*^*4*^ (BDSC:3410 and BDSC:25145), *tam*^*KO*^ (generated in [22]), BacTam (a gift from Hong Xu, National Heart, Lung and Blood Institute), *nos-Cas9* (BDSC:54591) and four lines carrying the *SM6a* balancer chromosome. All fly stocks were raised on standard media at 25°C unless otherwise stated.

Various heteroplasmic lines were generated via cytoplasmic transplantation as described in [5]. The stable *mt:yak*/*mt:ND2*^*del1*^+*CoI*^*T300I*^ was previously created by introducing cytoplasm of *D. yakuba* into the *mt:ND2*^*del1*^+*mt:CoI*^*T300I*^ embryos [8]. After the stable heteroplasmy was established, flies were balanced on the 2^nd^ or 3^rd^ chromosome by crossing to *Kr*^*If*^/*CyO* or *MKRS*/*TM6B* males, respectively. Once balanced, the flies were continuously backcrossed to *Kr*^*If*^/*CyO* or *MKRS*/*TM6B* males to maintain an isogenic nuclear background. To create *mt:ATP6[1]*/*mt:ND2*^*del1*^+*CoI*^*T300I*^ line, cytoplasm of *mt:ND2*^*del1*^+*CoI*^*T300I*^ was injected into *mt:ATP6[1]* embryos in order to create founder flies with a high percentage of *mt:ATP6[1]*. To generate *mt:ND2*^*del1*^/*mt:CoI*^*T300I*^*,* cytoplasm of *mt:ND2*^*del1*^ embryos was transferred to *mt:CoI*^*T300I*^ embryos to create heteroplasmic flies with a high percentage of *mt:CoI*^*T300I*^. To generate wild-type/*mt:ND2*^*del1*^*,* cytoplasm of wild-type embryos was transferred to *mt:ND2*^*del1*^ embryos to create heteroplasmic flies with a high percentage of *mt:ND2*^*del1*^. All the heteroplasmic lines were maintained and examined at 29°C, except the wild-type/*mt:ND2*^*del1*^ flies, which were maintained at 25°C instead.

### Method Details

#### The deficiency screen cross scheme

The screen was carried out at 29°C as shown in Figure 1B. Basically, for each deficiency, 5 males carrying the deletion chromosome were mated with 10 heteroplasmic females (generation 0) balanced with either *Kr*^*If*^/*CyO* (for 2^nd^ chromosome deficiencies) or *MKRS*/*TM6B* (for 3^rd^ chromosome deficiencies). After one generation, more than 10 female progeny (generation 1) with an individual deletion chromosome balanced by *CyO or TM6B* were mated with 10 *Kr*^*If*^/*CyO* or *MKRS*/*TM6B* males to maintain the deficiency and minimize variations in the nuclear background. Total DNA from 10 to 40 young male progeny (generation 2) that carry the deletion chromosome (balanced by *CyO* or *TM6B*) was extracted for qPCR analysis. Heterozygous mutants of *tam*, *pol* γ-β, *GatC*, and *twk* were tested with the same experimental setup for every generation. For controls, *Kr*^*If*^/*CyO* males were used instead of deficiency males for the first cross.

#### CRISPR/Cas9-based mutagenesis

CRISPR/Cas9-based mutagenesis was performed as described on FlyCRISRP (https://flycrispr.org/). In brief, two gRNAs were designed for each of the following genes: *pol* γ-β (gaaaaacgctggatgttgac, gctttgatgtttcagaagag), *GatC* (gcagctaacgcatcccacca, gatctggatttcggaggcgc), *twk* (tgctggcttacgtaaacaag, atatctgggcgatcgacggg), or *tam* (gtcacaatgtctcctacgac, ctacgacagggcgcgactga) using FlyCRISPR target finder (http://tools.flycrispr.molbio.wisc.edu/targetFinder/). Complimentary oligos were synthesized by Integrated DNA Technologies and were cloned into a pCFD5 plasmid. Plasmids were amplified and purified, and then injected into *nos-Cas9* flies (BDSC:54591) at a concentration of 200 ng/μl. Adults were then balanced by crossing to *Kr*^*If*^/*CyO* twice to establish individual stocks. The mutated sequences were verified by Sanger sequencing.

#### DNA extraction and quantitative PCR

Total DNA extraction was performed as described in [5]. In brief, 10 to 40 adult males were squashed in 500 μL of homogenization buffer (100 mM Tris-HCl (pH 8.8), 10 mM EDTA, 1% SDS) and incubated at 70°C for 30 min. Potassium acetate was added to a final concentration of 1 M, and samples were incubated on ice for 30 min. Samples were centrifuged at 20,000 g for 10 min at room temperature. DNA was recovered from the supernatant by adding 0.5x volume of isopropanol followed by washing with 70% ethanol. DNA was then dissolved in 100 μL Tris (10 mM, pH 8.0) before further dilution.

For all qPCR reactions, 2X SensiFast SYBR Green PCR Master Mix (Bioline 98020) was used in 20 μL reactions with 500 nM of each primer. For each reaction, 5% of a male’s total genomic DNA was used as the template to allow the Ct values to land between cycles 10-25. Each qPCR cycle was incubated at 95°C for 10 min followed by 35 cycles of 95°C for 30 s and 48°C for 30 s. Standard curves were plotted using a series of tenfold dilutions (2 × 10^7^ to 2 × 10^3^ copies per qPCR reaction) of the linearized PCR products containing regions covered by both the common and specific primer sets. The efficiency of each primer set was normalized by comparison to homoplasmic mtDNA that contain both the common and specific region. The absolute copy number of targeted regions was calculated according to the Ct value and the standard curve for one of the co-resident mtDNA genotypes (e.g., *mt:yak*, recognized by the specific primer set) and total mtDNA (recognized by the common primer set). All the primers are listed in Table S4.

#### Total RNA extraction and reverse transcription

Total RNA from 2-day old males was extracted based on the TRIzol reagent (Invitrogen) protocol. Ten males were ground with 750 μL of TRIzol reagent and incubated at room temperature for 10 min. Phenol was removed from samples by multiple rounds of chloroform extraction. RNA from the supernatant was precipitated by adding 0.5x isopropanol and washed once with 70% ethanol. The extracted RNA was then treated with RNase-free DNase I (New England Biolabs) for 30 min at 37°C to remove genomic DNA. Subsequently, DNase activity was heat-inactivated for 10 min at 65°C upon adding 1 μL of 50 mM EDTA. The RNA was then reverse-transcribed with Oligo (dT)_18_ primer using RevertAid First-strand cDNA synthesis kit (Invitrogen). The relative expression level of *tam* and *pol* γ-β was measured by qPCR and normalized to the expression level of house-keeping gene *Act42A* or *EF1*α. For each qPCR reaction, 2X SensiFast SYBR Green PCR Master mix (Bioline) was used in 20 μL reactions with 500 nM of each primer. The qPCR cycle was set as 95°C for 10 min followed by 35 cycles of 95°C for 30 s and elongation for 30 s. All the primers are listed in Table S4.

#### Embryo mtDNA extraction and copy number measurement

For each genotype, over 50 newly laid eggs (collected within 20 min after egg laying) were lysed in 100 μL of QuickExtract buffer (Lucigen, Thermo Fisher Scientific) in Precellys homogenizer. In brief, samples were agitated 3 times at 4,000 rpm for 30 s with a 30 s pause at room temperature. The homogenized samples were then incubated for 15 min at 65°C followed by 5 min at 95°C. The total mtDNA copy number was then measured by qPCR using the common primer set that binds to a conserved mtDNA region of *mt:yak* and *mt:ND2*^*del1*^+*CoI*^*T300I*^ (Table S4).

### Quantification and Statistical Analysis

As specified in all figure legends, the percentage of a given mitochondrial genotype in a heteroplasmic line (Figures 1C, 2A, 3, 4A, 4B, and S3C) was measured in at least three independent biological replicates. Each replicate contained 10-40 young adult males. Similarly, the total mtDNA copy number (Figure S3B) and the mRNA level of *tam* and *pol* γ-β genes (Figures S3A and S4B) were measured in three independent biological replicates. For mtDNA copy number quantifications, 50 newly laid eggs or ten 2-day old adult males were used for each replicate. For mRNA level quantifications, each replicate included ten 2-day old adult males. Figures are all presented as mean ± SD. All the statistical analyses were performed using GraphPad Prism 7.0. Differences were examined by unpaired Student’s t test. Significance was defined by ^∗^p < 0.05, ^∗∗^p < 0.005, and ^∗∗∗^p < 0.0005.

### Data and Code Availability

This study did not generate datasets and codes.

## Supplemental Information

Document S1. Figures S1–S4 and Tables S1–S4

Document S2. Article plus Supplemental Information
