# Supplementary material for: A Genome-wide Screen Reveals that Reducing Mitochondrial DNA Polymerase Can Promote Elimination of Deleterious Mitochondrial Mutations
Source: Curr Biol. 2019 Dec 16;29(24):4330–4336.e3. doi: 10.1016/j.cub.2019.10.060 (PMC6926476; doi:10.1016/j.cub.2019.10.060)
Supplement: Document S1. Figures S1–S4 and Tables S1–S4 [file mmc1.pdf]

**Current Biology, Volume *29***

## Supplemental Information

# A Genome-wide Screen Reveals that Reducing Mitochondrial DNA Polymerase Can Promote Elimination of Deleterious Mitochondrial Mutations

**Ason C.-Y. Chiang, Eleanor McCartney, Patrick H. O'Farrell, and Hansong Ma**

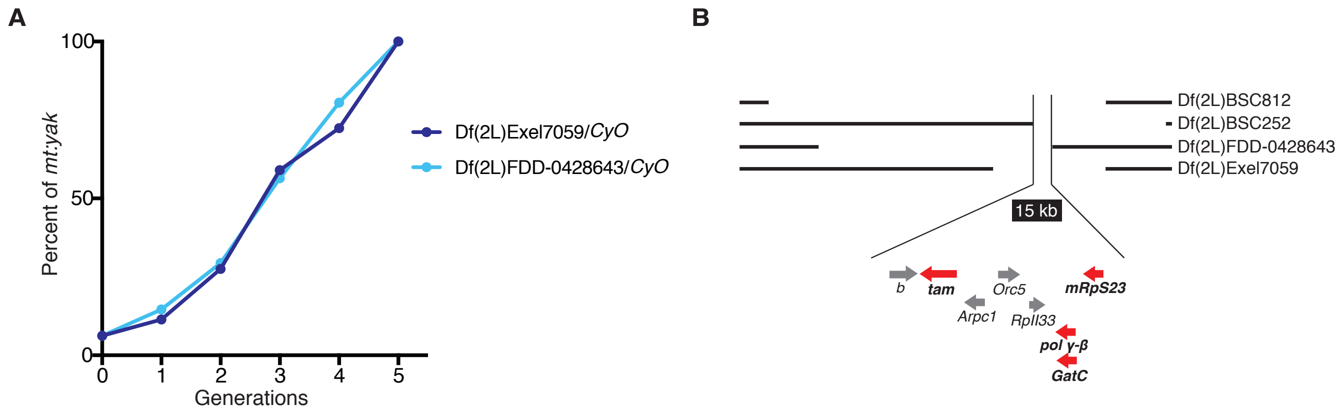


**Figure S1. Two additional deficiencies that cover the *tam* genomic region showed an increased *mt:yak* percentage over generations. Related to Figure 1.**

**A)** In Df(2L)Exel7059 and Df(2L)FDD-0428643 lines, the *mt:yak* took over after five generations. **B)** The common region deleted by the four deficiencies that similarly modified the heteroplasmy dynamic is indicated. It is 15 kb in size and contains eight genes, four of which encode mitochondrial proteins (red).

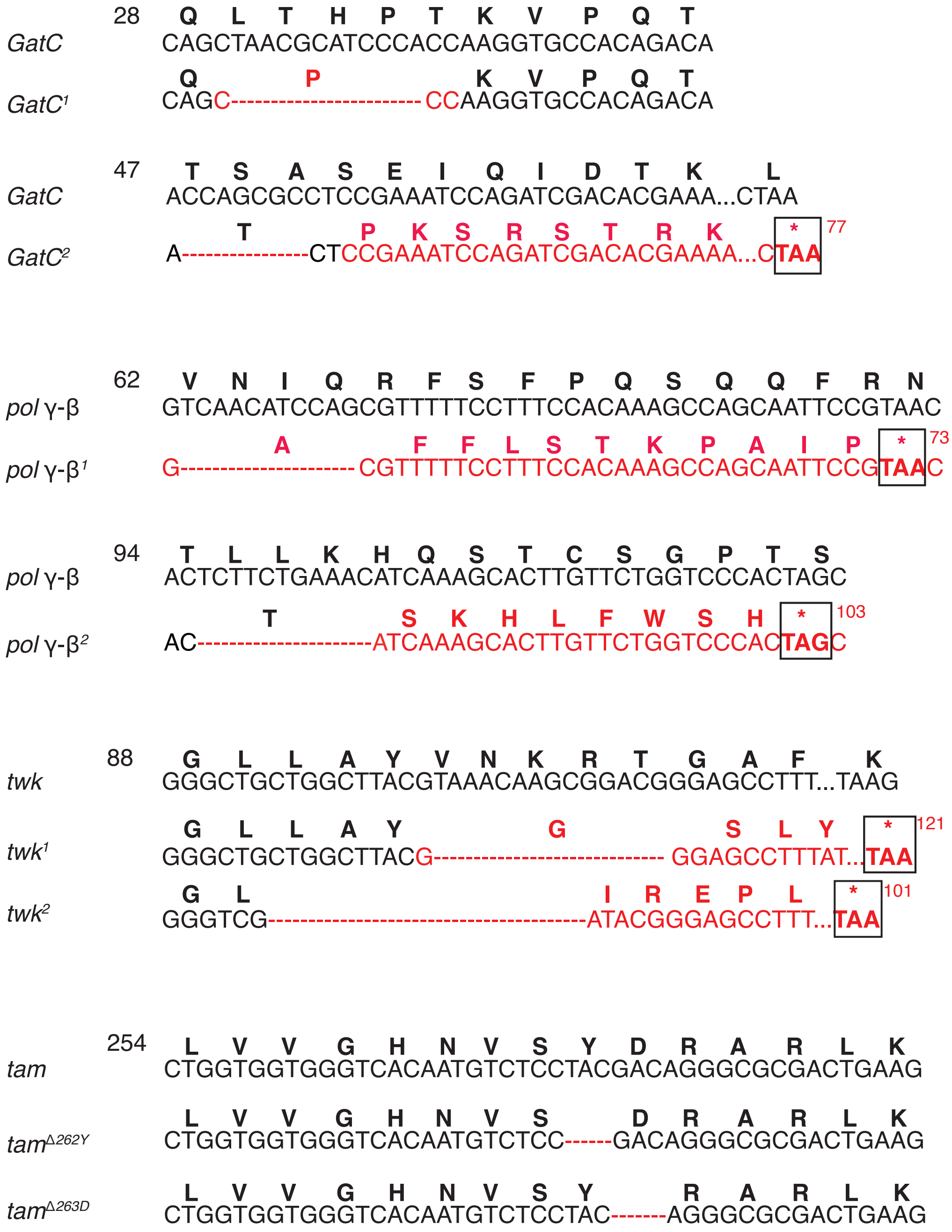


**Figure S2. Sequence details of *GatC*, *pol* γ-β, *twk* and *tam* mutants generated by CRISPR/Cas9-based editing. Related to Figures 2, 4, S3 and S4.**
Wild-type DNA and amino acid sequences (bold) are displayed in parallel to the mutant sequences with deletions (hyphen lines) that lead to frameshift changes in amino acid sequence (red) and a stop codon at the position indicated (asterisk, number of amino acid residues incorporated). The lethal phase of homozygote *twk* and *pol* γ-β mutants was in early pupation, whereas homozygous *GatC* mutants only reach late embryonic or early 1st instar larval stages. The *GatC* mutants were trans-heterozygous viable when crossed to the two *pol* γ-β mutants, indicating that the isolated mutants affected the targeted gene without inactivating its co-transcribed partner. Each *tam* mutant carries a 3 bp deletion, which removes a single amino acid at the highly conserved exonuclease domain. Both are homozygous viable but female sterile. For the amino acid residue 263, a D to A mutation has been previously reported to increase mtDNA mutation load over generations in *Drosophila* [S1,S2].

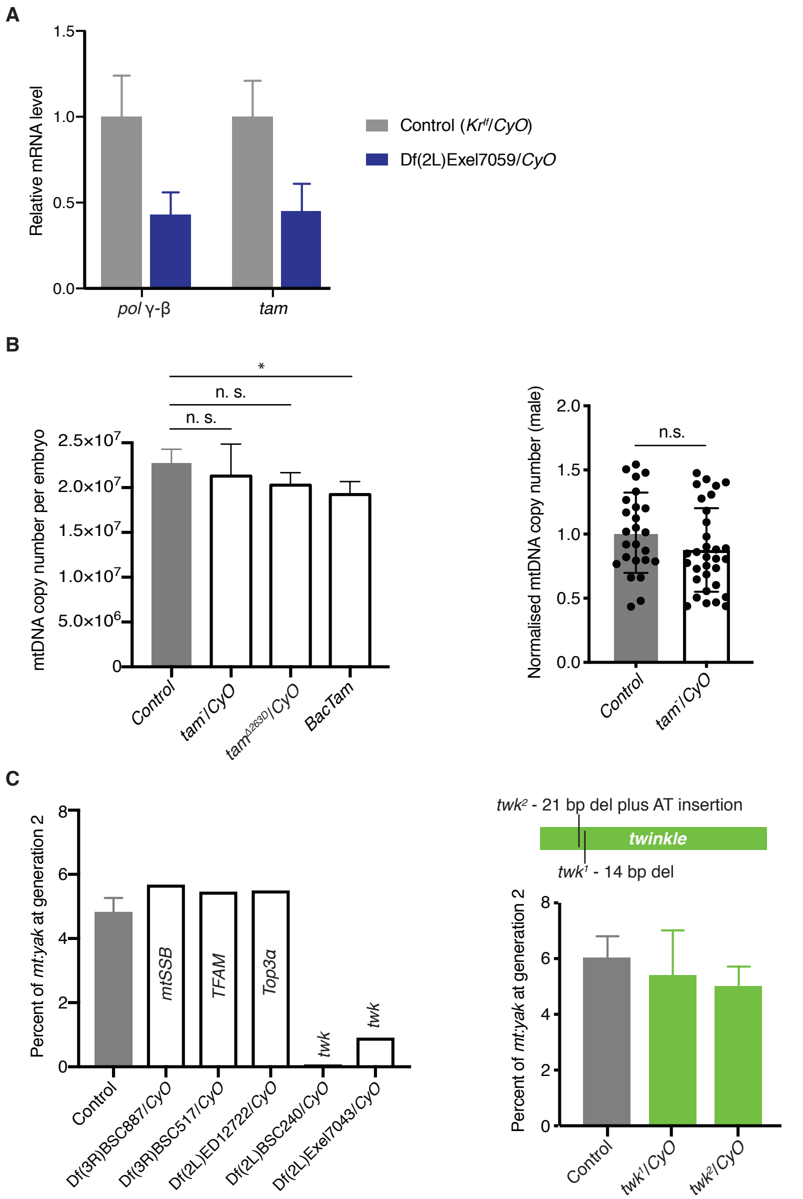


**Figure S3. Characterisation of different deficiencies and heterozygous mutants to assess the impact of replication proteins on the total mtDNA copy number and mtDNA competition. Related to Figure 2.**

**A)** The relative mRNA levels of *tam* and *pol γ-β* in adult male of Df(2L)Exel7059 were about half compared to those of *Kr*$^{If}$/*CyO*. Each sample represents an independent RNA extraction from a group of ten 2-day old adult males (n=3, Student's *t*-test, error bars: SDs). **B)** The heterozygous *tam* mutants (*tam*$^{3}$, *tam*$^{4}$, *tam*$^{KO}$ and *tam*$^{\Delta 263D}$) have a similar total mtDNA copy number compared to *Kr*$^{If}$/*CyO* flies. Left panel: the absolute mtDNA copy number per egg was measured in newly laid eggs by qPCR (n=3, *p*-value: Student's *t*-test, error bars: SDs). Each sample represents an extraction from a group of more than 50 eggs. Right panel: The mtDNA copy number per adult male was measured by qPCR and then normalized to the input amount of DNA. Each sample represents an extraction from a group of ten 2-day old adult males (n>25, *p*-value: Student's *t*-test, error bars: SDs). **C)** Other components of mtDNA replication machinery show no dosage-dependent impact on mtDNA competition. Left panel: the abundance of *mt:yak* was not changed in deficiency lines that delete one genomic copy of *mtSSB*, *TFAM* or *Top3α*, but was reduced in deficiencies removing one copy of *twk*. Right panel: heterozygous *twk* mutants showed no change in the *mt:yak* percentage in two generations. The two *twk* mutants were isolated by CRISPR/Cas9-based editing (Figure S2, error bars: SDs).

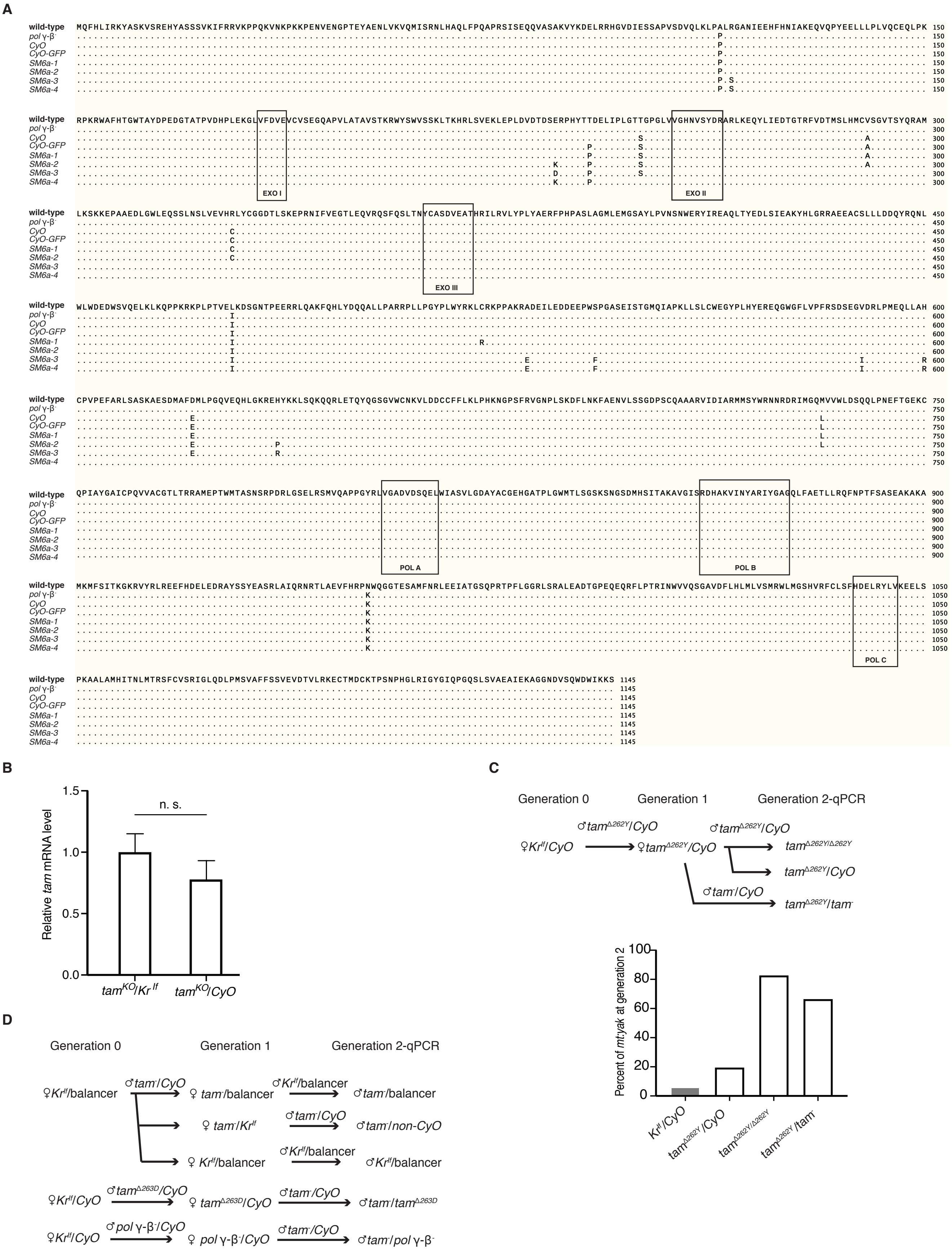
A
wild-type
pol γ-β-
CyO
CyO-GFP
SM6a-1
SM6a-2
SM6a-3
SM6a-4
EXO I
EXO II
EXO III
POL A
POL B
POL C
B
n. s.
Relative tam mRNA level
tamKO/Kr If
tamKO/CyO
C
Generation 0
Generation 1
Generation 2-qPCR
♀Krif/CyO
♂tamΔ262Y/CyO
♀tamΔ262Y/CyO
♂tamΔ262Y/CyO
♂tam-/CyO
tamΔ262Y/Δ262Y
tamΔ262Y/CyO
tamΔ262Y/tam-
Percent of mt:yak at generation 2
Krif/CyO
tamΔ262Y/CyO
tamΔ262Y/Δ262Y
tamΔ262Y/tam-
D
Generation 0
Generation 1
Generation 2-qPCR
♀Krif/balancer
♂tam-/CyO
♀ tam-/balancer
♂Krif/balancer
♂tam-/balancer
♀ tam-/Krif
♂tam-/CyO
♂tam-/non-CyO
♀ Krif/balancer
♂Krif/balancer
♂Krif/balancer
♀Krif/CyO
♂tamΔ263D/CyO
♀ tamΔ263D/CyO
♂tam-/CyO
♂tam-/tamΔ263D
♀Krif/CyO
♂pol γ-β-/CyO
♀ pol γ-β-/CyO
♂tam-/CyO
♂tam-/pol γ-β-

**Figure S4. Modulating Tam function alone is sufficient to influence competition between mitochondrial genomes. Related to Figure 4.**

**A)** The *tam* sequence for *CyO* related or unrelated 2nd chromosomes. The detailed lineages of the *CyO* related chromosomes are uncertain and the sequences appear to be at odds with a shared heritage among *SM6a* chromosomes. Flies carrying *SM6a-2*, *SM6a-3* and *SM6a-4* were obtained from the Bloomington *Drosophila* Stock Centre, and their stock numbers are 25166, 8045 and 27383, respectively. The amino acids that do not differ from the wild-type allele are indicated by dots. The three conserved exonuclease and polymerase domains are labelled (refer to [S3]). **B)** The relative mRNA level of *tam* in $tam^{KO}$ heterozygous with $Kr^{If}$ or *CyO* balancer chromosomes. Each sample represents an independent RNA extraction from a group of ten 2-day old adult males (n=3, Student's *t*-test, error bars: SDs). **C)** Homozygous or transheterozygous viable *tam* mutants revealed that reducing functional Tam alone is sufficient to increase the *mt:yak* percentage. The top panel illustrates the cross scheme used to introduce various *tam* alleles into the stable heteroplasmic line. The *mt:yak* percentage was measured in $tam^{\Delta 262Y}/CyO$, $tam^{\Delta 262Y}/tam^{\Delta 262Y}$ and $tam^{\Delta 262Y}/tam^{-}$ ($tam^{KO}$) adult males. **D)** The cross scheme to generate $tam^{-}$ heterozygotes with *CyO* related or unrelated 2nd chromosomes used in Figure 4A.

**Table S1. A list of all deficiencies tested in this study. Related to Figure 1.**

| | | | | |
|---|---|---|---|---|
| Df(2L)al | Df(2L)ED441 | Df(2R)BSC702 | Df(3L)BSC419 | Df(3R)BSC633 |
| Df(2L)ast2 | Df(2L)ED4651 | Df(2R)BSC769 | Df(3L)BSC449 | Df(3R)BSC650 |
| Df(2L)BSC106 | Df(2L)ED489 | Df(2R)BSC780 | Df(3L)BSC671 | Df(3R)BSC677 |
| Df(2L)BSC107 | Df(2L)ED50001 | Df(2R)BSC782 | Df(3L)BSC673 | Df(3R)BSC681 |
| Df(2L)BSC109 | Df(2L)ED5878 | Df(2R)BSC784 | Df(3L)BSC730 | Df(3R)BSC728 |
| Df(2L)BSC110 | Df(2L)ED629 | Df(2R)BSC787 | Df(3L)BSC774 | Df(3R)BSC738 |
| Df(2L)BSC111 | Df(2L)ED678 | Df(2R)BSC821 | Df(3L)BSC775 | Df(3R)BSC741 |
| Df(2L)BSC142 | Df(2L)ED690 | Df(2R)BSC865 | Df(3L)BSC797 | Df(3R)BSC748 |
| Df(2L)BSC143 | Df(2L)ED761 | Df(2R)BSC880 | Df(3L)BSC800 | Df(3R)BSC749 |
| Df(2L)BSC145 | Df(2L)ED775 | Df(2R)BSC883 | Df(3L)BSC815 | Df(3R)BSC750 |
| Df(2L)BSC148 | Df(2L)ED7853 | Df(2R)BSC885 | Df(3L)BSC816 | Df(3R)BSC790 |
| Df(2L)BSC149 | Df(2L)ED793 | Df(2R)BSC889 | Df(3L)BSC845 | Df(3R)BSC793 |
| Df(2L)BSC151 | Df(2L)ED8142 | Df(2R)CX1 | Df(3L)BSC884 | Df(3R)BSC819 |
| Df(2L)BSC159 | Df(2L)ED94 | Df(2R)ED1612 | Df(3L)ED201 | Df(3R)BSC874 |
| Df(2L)BSC165 | Df(2L)Exel6005 | Df(2R)ED1673 | Df(3L)ED208 | Df(3R)BSC887 |
| Df(2L)BSC169 | Df(2L)Exel6009 | Df(2R)ED1715 | Df(3L)ED210 | Df(3R)ED10639 |
| Df(2L)BSC17 | Df(2L)Exel6011 | Df(2R)ED1725 | Df(3L)ED217 | Df(3R)ED10642 |
| Df(2L)BSC172 | Df(2L)Exel6012 | Df(2R)ED1742 | Df(3L)ED229 | Df(3R)ED10845 |
| Df(2L)BSC180 | Df(2L)Exel6038 | Df(2R)ED1770 | Df(3L)ED230 | Df(3R)ED2 |
| Df(2L)BSC188 | Df(2L)Exel6277 | Df(2R)ED1791 | Df(3L)ED4196 | Df(3R)ED50003 |
| Df(2L)BSC204 | Df(2L)Exel7011 | Df(2R)ED2219 | Df(3L)ED4287 | Df(3R)ED5100 |
| Df(2L)BSC208 | Df(2L)Exel7034 | Df(2R)ED2247 | Df(3L)ED4293 | Df(3R)ED5147 |
| Df(2L)BSC209 | Df(2L)Exel7070 | Df(2R)ED2354 | Df(3L)ED4341 | Df(3R)ED5156 |
| Df(2L)BSC213 | Df(2L)Exel8038 | Df(2R)ED2426 | Df(3L)ED4421 | Df(3R)ED5177 |
| Df(2L)BSC214 | Df(2L)J39 | Df(2R)ED2457 | Df(3L)ED4457 | Df(3R)ED5330 |
| Df(2L)BSC227 | Df(2L)lt109 | Df(2R)ED2487 | Df(3L)ED4470 | Df(3R)ED5339 |
| Df(2L)BSC233 | Df(2L)M24F-B | Df(2R)ED2747 | Df(3L)ED4475 | Df(3R)ED5428 |
| Df(2L)BSC240 | Df(2L)r10 | Df(2R)ED3385 | Df(3L)ED4486 | Df(3R)ED5474 |
| Df(2L)BSC241 | Df(2L)tkv3 | Df(2R)ED3610 | Df(3L)ED4502 | Df(3R)ED5514 |
| Df(2L)BSC244 | Df(2R)14H10W-35 | Df(2R)ED3683 | Df(3L)ED4543 | Df(3R)ED5577 |
| Df(2L)BSC252 | Df(2R)BSC132 | Df(2R)ED3728 | Df(3L)ED4674 | Df(3R)ED5578 |
| Df(2L)BSC256 | Df(2R)BSC135 | Df(2R)ED3791 | Df(3L)ED4710 | Df(3R)ED5623 |
| Df(2L)BSC277 | Df(2R)BSC152 | Df(2R)ED50004 | Df(3L)ED4858 | Df(3R)ED5644 |
| Df(2L)BSC278 | Df(2R)BSC161 | Df(2R)Exel6061 | Df(3L)ED4978 | Df(3R)ED5705 |
| Df(2L)BSC291 | Df(2R)BSC19 | Df(2R)Exel6062 | Df(3L)ED50002 | Df(3R)ED5718 |
| Df(2L)BSC292 | Df(2R)BSC199 | Df(2R)Exel6064 | Df(3L)ED5017 | Df(3R)ED5780 |
| Df(2L)BSC295 | Df(2R)BSC267 | Df(2R)Exel6066 | Df(3L)Exel6085 | Df(3R)ED5815 |
| Df(2L)BSC354 | Df(2R)BSC273 | Df(2R)Exel6069 | Df(3L)Exel6109 | Df(3R)ED5938 |
| Df(2L)BSC37 | Df(2R)BSC274 | Df(2R)Exel6284 | Df(3L)Exel6112 | Df(3R)ED6025 |
| Df(2L)BSC454 | Df(2R)BSC280 | Df(2R)Exel7130 | Df(3L)Exel6132 | Df(3R)ED6085 |
| Df(2L)BSC455 | Df(2R)BSC281 | Df(2R)Exel7149 | Df(3L)M21 | Df(3R)ED6096 |
| Df(2L)BSC50 | Df(2R)BSC298 | Df(2R)Exel7162 | Df(3L)ZN47 | Df(3R)ED6220 |

| | | | | |
|---|---|---|---|---|
| Df(2L)BSC6 | Df(2R)BSC303 | Df(2R)Exel8057 | Df(3R)10-65 | Df(3R)ED6232 |
| Df(2L)BSC688 | Df(2R)BSC305 | Df(2R)Kr10 | Df(3R)A113 | Df(3R)ED6255 |
| Df(2L)BSC689 | Df(2R)BSC307 | Df(2R)M41A10 | Df(3R)Antp17 | Df(3R)ED6280 |
| Df(2L)BSC690 | Df(2R)BSC308 | Df(2R)M60E | Df(3R)BSC137 | Df(3R)ED6346 |
| Df(2L)BSC692 | Df(2R)BSC331 | Df(2R)X1 | Df(3R)BSC140 | Df(3R)ED6361 |
| Df(2L)BSC781 | Df(2R)BSC347 | Df(2R)X58-12 | Df(3R)BSC141 | Df(3R)ED7665 |
| Df(2L)BSC812 | Df(2R)BSC355 | Df(3L)1-16 | Df(3R)BSC321 | Df(3R)Exel6154 |
| Df(2L)BSC892 | Df(2R)BSC356 | Df(3L)6B-29+Df(3R)6B-29 | Df(3R)BSC43 | Df(3R)Exel6155 |
| Df(2L)C144 | Df(2R)BSC361 | Df(3L)AC1 | Df(3R)BSC464 | Df(3R)Exel6159 |
| Df(2L)dpp[d14] | Df(2R)BSC383 | Df(3L)Aprt-32 | Df(3R)BSC469 | Df(3R)Exel6196 |
| Df(2L)drm-P2 | Df(2R)BSC425 | Df(3L)BSC113 | Df(3R)BSC47 | Df(3R)Exel6197 |
| Df(2L)ed1 | Df(2R)BSC427 | Df(3L)BSC117 | Df(3R)BSC476 | Df(3R)Exel6201 |
| Df(2L)ED105 | Df(2R)BSC429 | Df(3L)BSC119 | Df(3R)BSC489 | Df(3R)Exel6202 |
| Df(2L)ED1102 | Df(2R)BSC485 | Df(3L)BSC181 | Df(3R)BSC497 | Df(3R)Exel6203 |
| Df(2L)ED1203 | Df(2R)BSC550 | Df(3L)BSC220 | Df(3R)BSC501 | Df(3R)Exel6264 |
| Df(2L)ED1272 | Df(2R)BSC595 | Df(3L)BSC224 | Df(3R)BSC502 | Df(3R)Exel6270 |
| Df(2L)ED1315 | Df(2R)BSC597 | Df(3L)BSC27 | Df(3R)BSC503 | Df(3R)Exel6272 |
| Df(2L)ED136 | Df(2R)BSC598 | Df(3L)BSC289 | Df(3R)BSC504 | Df(3R)Exel7328 |
| Df(2L)ED1378 | Df(2R)BSC599 | Df(3L)BSC368 | Df(3R)BSC507 | Df(3R)Exel7378 |
| Df(2L)ED1473 | Df(2R)BSC604 | Df(3L)BSC371 | Df(3R)BSC515 | Df(3R)FDD-031795 |
| Df(2L)ED19 | Df(2R)BSC608 | Df(3L)BSC375 | Df(3R)BSC517 | Df(3R)P115 |
| Df(2L)ED247 | Df(2R)BSC630 | Df(3L)BSC388 | Df(3R)BSC547 | Df(3R)R133 |
| Df(2L)ED250 | Df(2R)BSC651 | Df(3L)BSC389 | Df(3R)BSC549 | Df(3R)Tpl10 |
| Df(2L)ED3 | Df(2R)BSC661 | Df(3L)BSC391 | Df(3R)BSC567 | Df(3R)Ubx109 |
| Df(2L)ED334 | Df(2R)BSC664 | Df(3L)BSC411 | Df(3R)BSC619 | Df(3R)X3F |
| Df(2L)ED385 | Df(2R)BSC701 | Df(3L)BSC414 | Df(3R)BSC621 | |

Deficiencies are organised in alphabetical order, and ones that generated no, few or sick progeny when crossed to the stable heteroplasmic females are underlined.

**Table S2. A deficiency screen identified 38 nuclear loci carrying genes that affect mtDNA competition. Related to Figure 1.**

| Deficiencies | Total tested | Sick lines | *mt:yak* ≤ 2% | *mt:yak* ≥ 10% |
|---|---|---|---|---|
| 2nd chromosome | 184 | 25 | 30 | 3 |
| 3rd chromosome | 155 | 38 | 3 | 2 |
| Total | 339 | 63 | 33 | 5 |

**Table S3. A list of deficiencies that reduced *mt:yak* to ≤ 2%, increased *mt:yak* to ≥ 10%. Related to Figure 1.**

| Deficiency | Percent of *mt:yak* | Deficiency | Percent of *mt:yak* |
|---|---|---|---|
| *mt:yak* ≤ 2% | | | |
| Df(2L)BSC107 | 1.3 | Df(2R)BSC280 | 1.85±0.32 |
| Df(2L)BSC110 | 1.67±0.38 | Df(2R)BSC361 | 1.78±0.11 |
| Df(2L)BSC111 | 1.86±0.82 | Df(2R)BSC383 | 1.44±1.23 |
| Df(2L)BSC169 | 1.59±0.14 | Df(2R)BSC425 | 1.44±0.16 |
| Df(2L)BSC204 | 1.61±0.52 | Df(2R)BSC597 | 1.60±0.55 |
| Df(2L)BSC256 | 1.62±0.35 | Df(2R)BSC630 | 1.94±0.13 |
| Df(2L)BSC455 | 1.71±0.51 | Df(2R)BSC769 | 1.55±0.26 |
| Df(2L)BSC689 | 2.01±0.01 | Df(2R)BSC880 | 1.23±0.92 |
| Df(2L)BSC692 | 2.02±0.01 | Df(2R)ED1725 | 1.11 |
| Df(2L)ED678 | 1.93±0.14 | Df(2R)ED1791 | 1.56±0.39 |
| Df(2L)Exel6005 | 1.99 | Df(2R)Exel6061 | 1.92±0.11 |
| Df(2L)Exel7070 | 1.35±0.51 | Df(3R)Exel6159 | 1.82 |
| Df(2R)BSC267 | 1.64 | Df(3R)Exel6264 | 2.00 |
| Df(2R)BSC273 | 2.11±0.91 | Df(3R)Exel6272 | 1.36 |
| *mt:yak* ≤ 1% | | | |
| Df(2L)BSC149 | 0.66±0.68 | Df(2R)BSC651 | 0.91±0.31 |
| Df(2L)BSC240 | 0.03±0.03 | Df(2R)BSC699 | 0.34±0.18 |
| Df(2L)BSC241 | 0.44±0.38 | | |
| *mt:yak* ≥ 10% | | | |
| Df(2L)BSC252 | 29.96±3.16 | Df(3R)Tpl10 | 12.74 |
| Df(2L)BSC812 | 26.25±2.46 | Df(3L)BSC730 | 10.14 |
| Df(2L)C144 | 12.91±3.93 | | |

All the deficiencies were tested at least twice. For those tested by three independent experiments, the percentage of *mt:yak* is presented as mean ± SD.

**Table S4. Primers used in this study. Related to STAR Methods.**

| Primers for qPCR | | |
|---|---|---|
| Heteroplasmy line | Common primer set | Specific primer set |
| ***mt:yak***/*mt:ND2*$^{del1}$+*CoI*$^{T300I}$ | cttttaatggttaaattccatttata<br>ttattattacaatgaaaatgtaaggt | aaatcatattgaacctaaaaataatg<br>attctttgaataaatctctattaaat |
| ***mt:ATP6[1]***/*mt:ND2*$^{del1}$+*CoI*$^{T300I}$ | aattttatcagttattattggagc<br>tcaattaatcatttagggtgaatatt | gtaaagaagtttgatttaatcc<br>tgattctctaattattaaagatctt |
| *mt:ND2*$^{del1}$/***mt:CoI***$^{T300I}$ and<br>**wild-type**/*mt:ND2*$^{del1}$ | aattttatcggttattattggagc<br>gtaaagaagtttgatttagtcc | tcaattaatcatttaggatgaatatt<br>tgattctctaattattaaagatctt |

| Primers for RT-qPCR | | |
|---|---|---|
| Gene | Primer set | Elongation temperature |
| *Act42A* | caccatgaagattaagattgttg<br>aacagagtacttgcgttc | 53°C |
| *EF1*$\alpha$ | gcgtgggtttgtgatcagtt<br>gatcttctccttgcccatcc | 60°C |
| *tam* | tggaggacagggcctacag<br>tcttccaggcgattgaacatg | 60°C |
| *pol* $\gamma$-$\beta$ | aaccgactgtcataaggt<br>gtgtaagagcagggattg | 53°C |

The mitochondrial genotype recognised by the specific primer set in each heteroplasmic line is highlighted in bold.

## Supplemental References


S1 Bratic A, Kauppila TES, Macao B, Groenke S, Siibak T, Stewart JB, et al. Complementation between polymerase- and exonuclease-deficient mitochondrial DNA polymerase mutants in genomically engineered flies. Nat Commun 2015;6. doi:10.1038/ncomms9808.

S2 Samstag CL, Hoekstra JG, Huang C-H, Chaisson MJ, Youle RJ, Kennedy SR, et al. Deleterious mitochondrial DNA point mutations are overrepresented in *Drosophila* expressing a proofreading-defective DNA polymerase gamma. PLoS Genet 2018;14. doi:10.1371/journal.pgen.1007805.

S3 Kaguni LS, DNA polymerase gamma, the mitochondrial replicase, Annual Review of Biochemistry 2004 (73):293-320
