# Supplementary material for: A Genome-wide Screen Reveals that Reducing Mitochondrial DNA Polymerase Can Promote Elimination of Deleterious Mitochondrial Mutations
Source: Curr Biol. 2019 Dec 16;29(24):4330–4336.e3. doi: 10.1016/j.cub.2019.10.060 (PMC6926476; doi:10.1016/j.cub.2019.10.060)
Supplement: Document S2. Article plus Supplemental Information [file mmc2.pdf]

Report

Current Biology

A Genome-wide Screen Reveals that Reducing Mitochondrial DNA Polymerase Can Promote Elimination of Deleterious Mitochondrial Mutations

Graphical Abstract

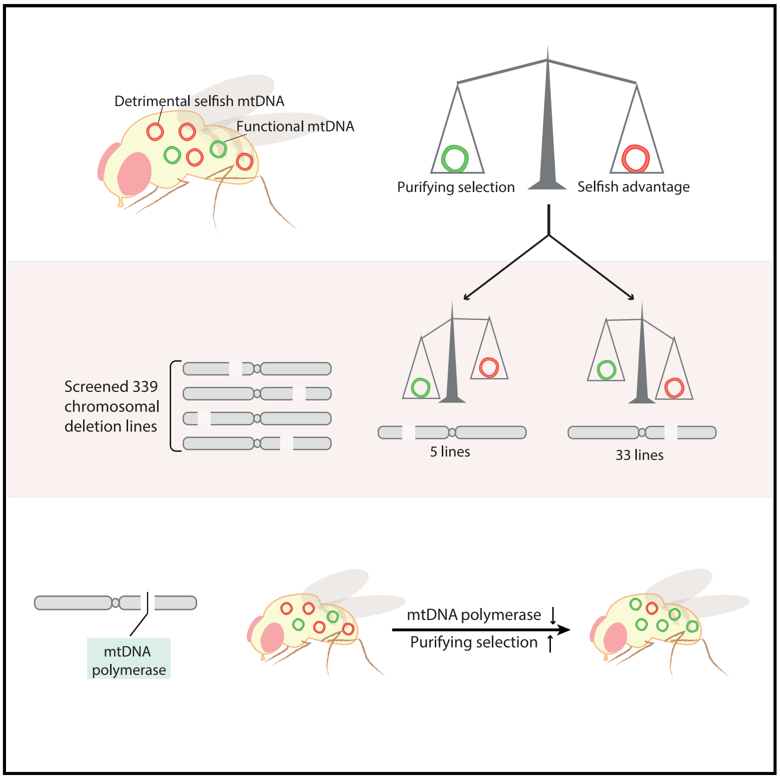

Highlights

- Multiple nuclear factors affect selective transmission of mitochondrial genomes
- Reducing mtDNA polymerase restricts the transmission of detrimental mtDNA mutants

Authors

Ason C.-Y. Chiang, Eleanor McCartney, Patrick H. O'Farrell, Hansong Ma

Correspondence

ofarrell@cgl.ucsf.edu (P.H.O.), hm555@cam.ac.uk (H.M.)

In Brief

Mitochondrial genomes within an organism compete for transmission. Chiang et al. reveal that multiple nuclear genes can affect this competition. In particular, reducing the nuclear-encoded mtDNA polymerase limits the transmission of detrimental mtDNA mutants. Thus, the nuclear genome can affect mitochondrial disease progression and mtDNA evolution.

Chiang et al., 2019, Current Biology 29, 4330–4336
December 16, 2019 © 2019 The Author(s). Published by Elsevier Ltd.
https://doi.org/10.1016/j.cub.2019.10.060

Current Biology
Report

A Genome-wide Screen Reveals that Reducing Mitochondrial DNA Polymerase Can Promote Elimination of Deleterious Mitochondrial Mutations

Ason C.-Y. Chiang,[1,2] Eleanor McCartney,[1,2] Patrick H. O'Farrell,[3,*] and Hansong Ma[1,2,4,*]
[1]Wellcome Trust/Cancer Research UK Gurdon Institute, Tennis Court Road, Cambridge CB2 1QN, UK
[2]Department of Genetics, University of Cambridge, Downing Street, Cambridge CB2 3EH, UK
[3]Department of Biochemistry and Biophysics, University of California, San Francisco, San Francisco, CA 94143, USA
[4]Lead Contact
*Correspondence: ofarrell@cgl.ucsf.edu (P.H.O.), hm555@cam.ac.uk (H.M.)
https://doi.org/10.1016/j.cub.2019.10.060

SUMMARY

A mutant mitochondrial genome arising amid the pool of mitochondrial genomes within a cell must compete with existing genomes to survive to the next generation. Even weak selective forces can bias transmission of one genome over another to affect the inheritance of mitochondrial diseases and guide the evolution of mitochondrial DNA (mtDNA). Studies in several systems suggested that purifying selection in the female germline reduces transmission of detrimental mitochondrial mutations [1–7]. In contrast, some selfish genomes can take over despite a cost to host fitness [8–13]. Within individuals, the outcome of competition is therefore influenced by multiple selective forces. The nuclear genome, which encodes most proteins within mitochondria, and all external regulators of mitochondrial biogenesis and dynamics can influence the competition between mitochondrial genomes [14–18], yet little is known about how this works. Previously, we established a *Drosophila* line transmitting two mitochondrial genomes in a stable ratio enforced by purifying selection benefiting one genome and a selfish advantage favoring the other [8]. Here, to find nuclear genes that impact mtDNA competition, we screened heterozygous deletions tiling ~70% of the euchromatic regions and examined their influence on this ratio. This genome-wide screen detected many nuclear modifiers of this ratio and identified one as the catalytic subunit of mtDNA polymerase gene (*POLG*), *tam*. A reduced dose of *tam* drove elimination of defective mitochondrial genomes. This study suggests that our approach will uncover targets for interventions that would block propagation of pathogenic mitochondrial mutations.

RESULTS AND DISCUSSION

Drosophila melanogaster tolerates heterozygous deletions well, and collections of chromosomal deletions (i.e., deficiencies) tiling 98% of the euchromatic genome have been developed for genetic screening purposes [19, 20]. To uncover nuclear regions that regulate mitochondrial DNA (mtDNA) competition, we performed a deficiency screen on a heteroplasmic line that stably transmits two genomes: a functional mtDNA from *Drosophila yakuba* (*mt:yak*) and a temperature-sensitive lethal genome harboring two mutations, $mt{:}ND2^{del1}$+CoI^{T300I} from *D. melanogaster* (Figure 1A) [8]. Previously, we showed that the *D. melanogaster* genome has a selfish transmission advantage, whereas *mt:yak* is favored by purifying selection because it provides the functional *mt:CoI*. The two opposing selections are balanced so that the heteroplasmic ratio (~5% *mt:yak* to ~95% $mt{:}ND2^{del1}$+CoI^{T300I}) was stable for over 70 generations at the restrictive temperature (29°C) of the $mt{:}ND2^{del1}$+CoI^{T300I} genome (Figure 1A). We reasoned that a balance of two selective forces would be very sensitive to perturbation. Perhaps just a change in the gene dose of nuclear genes modulating competition would alter the heteroplasmic ratio.

For the screen, 339 deletion chromosomes covering most of chromosomes II and III were introduced into the heteroplasmic flies (Table S1). Sixty-three crosses produced no or few progeny carrying the deletion (Table S1). Although it is likely that the lethality for some of the crosses was due to an inability to maintain the functional *mt:yak* genome, here we focus only on the 276 lines that produced progeny; in these, we measured the *mt:yak* percentage in adult males one generation after the deletion was introduced (i.e., generation 2; Figure 1B). Five lines had a substantially higher percentage of *mt:yak* (≥ 10%), whereas 33 lines had a lower percentage (≤ 2%) (Tables S2 and S3). More than 10% of the tested lines changed the *mt:yak* percentage, leading us to conclude that multiple nuclear factors directly or indirectly regulate competition between mitochondrial genomes.

Of the 38 deficiencies that altered the *mt:yak* percentage, the two (BSC252 and BSC812) causing the largest increase (from ~5% to ~28%) partially overlap (Table S3; Figures 1C and 1D). This suggests that a locus lying in the 60 kb region of the 2nd chromosome removed by both deletions is responsible for the observed phenotype (Figure 1D). Consistent with a common defect, both deletions caused a progressive and parallel rise in *mt:yak* over multiple generations until it reached 100% at generation 5 (Figure 1C). To produce this progressive heritable rise, these deletions must give *mt:yak* a selective advantage in the germline allowing accumulation in each generation. To confirm and further narrow down the responsible region, we tested two more deletions: Exel7059 (lacking the entire 60 kb region) and

 © 2019 The Author(s). Published by Elsevier Ltd.
This is an open access article under the CC BY license (http://creativecommons.org/licenses/by/4.0/).

CellPress

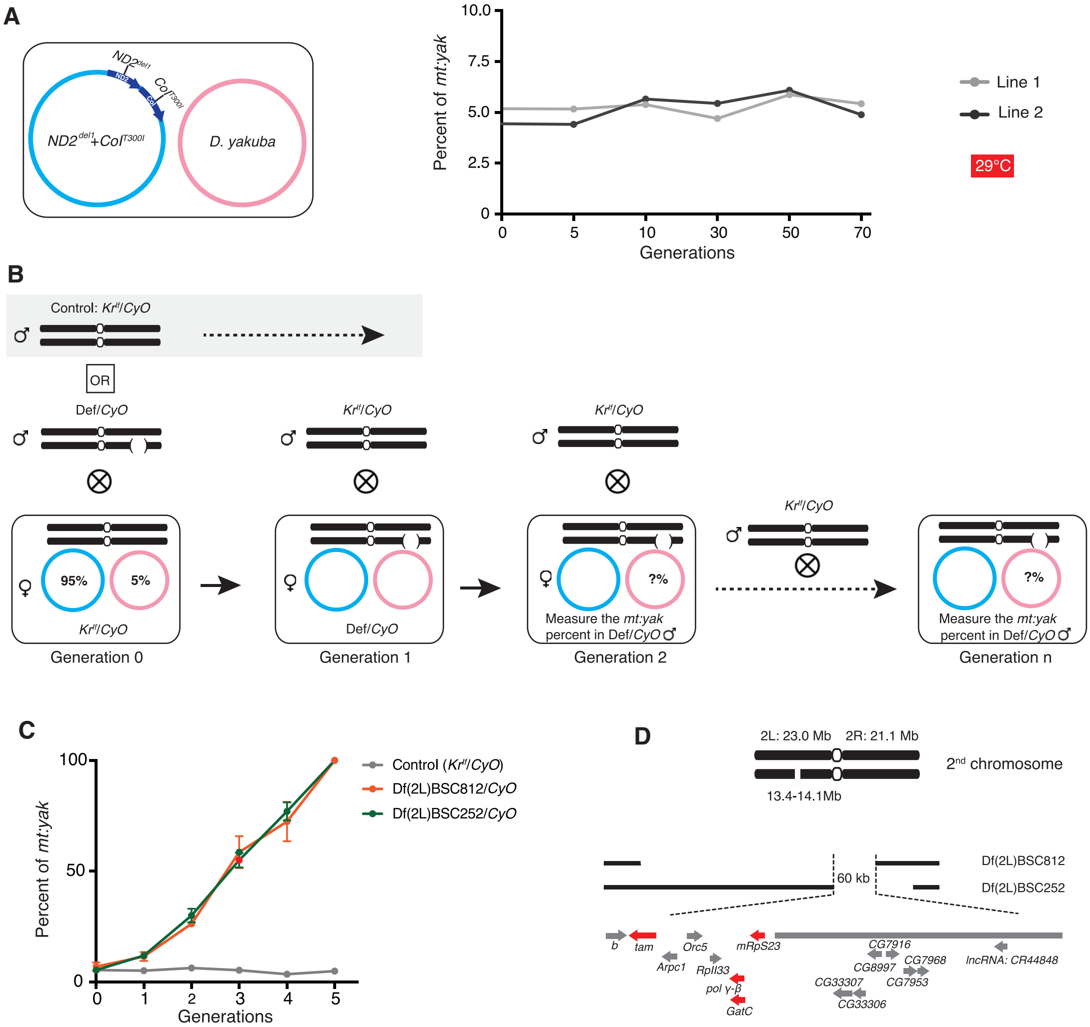

Figure 1. A Deficiency Screen Identified Two Overlapping Deletion Lines that Significantly Increased the Proportion of *mt:yak*

(A) The stable heteroplasmic line. The *D. melanogaster* mtDNA (blue circle) has two mutations, $ND2^{del1}$ and CoI^{T300I}. $ND2^{del1}$ is a 9-bp in-frame deletion in the gene encoding NADH-dehydrogenase 2 (dark blue); it is a viable hypomorphic allele. CoI^{T300I} is a temperature-sensitive allele of cytochrome *c* oxidase I (dark blue); when homoplasmic, it is lethal at 29°C but viable at lower temperatures. Although *mt:yak* (pink circle) is fully functional in *D. melanogaster*, it is usually outcompeted by the endogenous wild-type *D. melanogaster* mtDNA. However, when paired with *mt:*$ND2^{del1}$+CoI^{T300I}*,* *mt:yak* is stably transmitted at ~5% for over 70 generations at 29°C [8].

(B) The genetic cross scheme to introduce deletion chromosomes (Table S1) into the stable heteroplasmic line (only shown for the 2nd chromosome deficiencies schematized by a bracketed interruption). First, 10 Kr^{If}/*CyO* heteroplasmic females (generation 0) were crossed to 5 deficiency/*CyO* (Def/*CyO*) males to generate 10–20 female progeny with the genotype of Def/*CyO* (generation 1), which were further crossed to 10 Kr^{If}/*CyO* males to produce progeny (generation 2). Def/*CyO* males of generation 2 were collected to measure the *mt:yak* percentage via qPCR. For some deficiencies, Def/*CyO* males were collected at multiple generations to follow the *mt:yak* percentage over time. In controls, Kr^{If}/*CyO* males were used for the first cross instead of Def/*CyO* males. All the subsequent steps were the same, and Kr^{If}/*CyO* males of generation 2 were collected to measure the *mt:yak* percentage.

(C) Two overlapping deficiencies (balanced by *CyO*) increased *mt:yak* transgenerationally (see also Tables S2 and S3). The *mt:yak* percentage reached 100% after five generations. Error bars indicate SDs of three independent experiments.

(D) The region deleted in both deficiencies contains 15 genes, four of which encode mitochondrial proteins (red) (also refer to Figure S1).

FDD-0428643 (only lacking the left 15 kb segment of the 60 kb region). Both gave the same increase in the *mt:yak* percentage (Figure S1A). This confined our candidates to eight genes, including four with known mitochondrial functions: mtDNA polymerase catalytic subunit polymerase gene *POLG* (*tam*), mtDNA polymerase accessory subunit *POLG2* (*pol* γ-β), glutamyl tRNA-aminotransferase *GatC*, and mitochondrial ribosomal protein *mRpS23* (Figure S1B).

To investigate whether *pol* γ-β and its neighboring and co-transcribed gene *GatC* play a role in regulating mtDNA transmission, we generated loss-of-function mutants via CRISPR/Cas9-based editing (Figure S2). Heterozygous mutants of *pol* γ-β and *GatC* did not alter *mt:yak* percentages, suggesting neither was responsible for the changes in the heteroplasmic ratio (Figure 2A). We then tested three *tam* mutants: two classical mutations, tam^3 and tam^4, which contain small deletions that

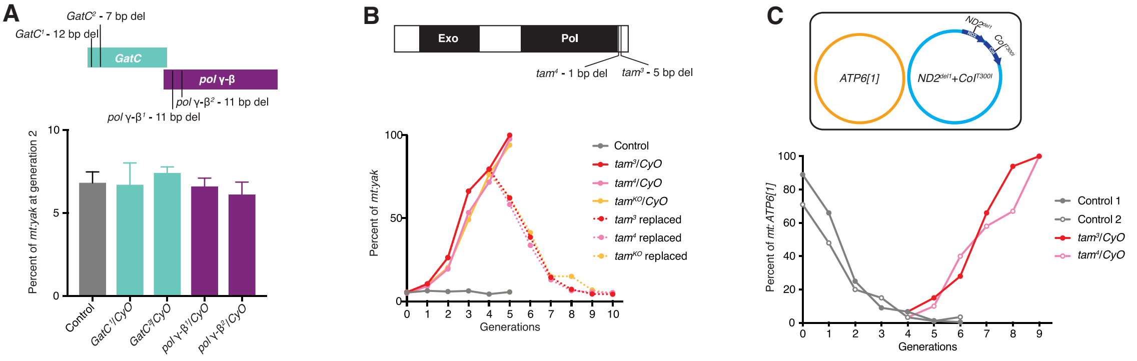

Figure 2. Reducing *tam* Gene Dose Increased the Abundance of Functional mtDNA in Two Heteroplasmic Lines

(A) Reducing the dose of *pol* γ-β or *GatC* showed no effect on mtDNA competition. Top: a schematic shows the genomic arrangement of the co-transcribed genes *GatC* and *pol* γ-β with the position and description of loss-of-function mutants isolated via CRISPR/Cas9-based editing (Figure S2). Bottom: the histogram shows the *mt:yak* percentage for different nuclear genotypes. Error bars indicate SDs of three independent experiments.

(B) Heterozygosity for any of the three mutant alleles of *tam* over the *CyO* chromosome dramatically shifted the heteroplasmic ratio (see Figure S3 for the total mtDNA copy number of the *tam* heterozygotes). Top: a schematic of Tam protein shows functional domains and the positions of two homozygous lethal mutations, tam^3 and tam^4. At 29°C, the *mt:yak* percentage increased in tam^3, tam^4, and tam^{KO} heterozygous mutants with a speed similar to that observed with the BSC252 and BSC812 deficiencies (Figure 1C). Chromosomes bearing *tam* mutations were introduced at generation 0 and the *mt:yak* percentage was followed over generations via qPCR (solid line). From generation 1 to 3, only progeny heterozygous for the *tam* mutation were followed and crossed to Kr^{If}/CyO to examine the cross-generational dose effect of *tam*. At generation 4, the female progeny lacking the *tam* mutation were also mated with Kr^{If}/CyO, and these populations were maintained in parallel for another 6 generations to assess the *mt:yak* percentage (dotted lines).

(C) Reducing *tam* dose also increased the percentage of the functional genome (*mt:ATP6[1]*) in the *mt:ATP6[1]/mt:ND2*^{del1}+*CoI*^{T300I} line. The percentage of *mt:ATP6[1]* in two heteroplasmic lineages (control 1 and control 2) was measured over generations at 29°C. At generation 4, female progeny were divided into two populations: one was mated with males heterozygous for tam^3 or tam^4 to remove one functional copy of *tam* and maintained in the heterozygous *tam* mutant background (balanced by *CyO*) for the subsequent generations; the other population was mated with Kr^{If}/CyO males as controls. All the controls refer to Kr^{If}/CyO.

shift the reading frame [21], and tam^{KO}, which removes the entire coding region including the UTRs [22]. Heterozygosity for each of these *tam* mutations increased the *mt:yak* percentage just as observed with the four deficiencies (Figure 2B). By generation 5, *mt:yak* took over. To test the reversibility of the effect, we restored a wild-type *tam* genotype in some flies having a residual ~20% of *mt:ND2*^{del1}+*CoI*^{T300I} at generation 4 and followed over subsequent generations (Figure 2B). In these flies, selection reversed its course and the *mt:yak* percentage declined. After four generations, *mt:yak* levels re-balanced at ~5%. Of note, in accord with the gene dose, the mRNA level of *tam* is halved in the deletion lines (Figure S3A). Despite the dramatic shifts in the relative abundance of the two genomes, only minor fluctuations in total mtDNA copy number were detected in the newly laid eggs and adult flies (Figure S3B; also shown in [22]). We conclude that removing one functional genomic copy of *tam* but not *pol* γ-β or *GatC* increased the *mt:yak* percentage in the given nuclear background.

We were somewhat surprised that the dose of the gene encoding the catalytic subunit of POLG modified competition whereas the dose of the essential accessory subunit did not. To pursue this further, we tested other genes involved in mtDNA replication (*twk*, *mtSSB*, *TFAM*, and *Top3α*) and did not detect modification of the heteroplasmic ratio in a manner dependent on the dose of these genes (Figure S3C). Thus, at least in the context of our screen, the input of *tam* appears to be relatively specific among replication functions.

To test whether the influence of *tam* dose on mtDNA competition extended beyond the specific mtDNA pair used in the screen, we examined a different pairing of mitochondrial genomes. We previously showed that when the *mt:ND2*^{del1}+*CoI*^{T300I} genome is paired with a distantly related *D. melanogaster* mitochondrial genome, *mt:ATP6[1]*, the temperature-sensitive mutant genome exhibits such a powerful selfish advantage that it overrides the constraint of purifying selection; the mutant genome replaced the complementing *mt:ATP6[1]* over a few generations, leading to the death of the entire lineage at 29°C [23]. We re-established two independent lineages of this unstable heteroplasmy, and *mt:ND2*^{del1}+*CoI*^{T300I} percentages rose rapidly as expected (Figure 2C). Before the demise of the stock, we removed one copy of functional *tam* by introducing chromosomes bearing the tam^3 or tam^4 mutation in flies at generation 4, which still had ~10% *mt:ATP6[1]*. Instead of observing a continued decline of *mt:ATP6[1]* as in control flies, *mt:ATP6[1]* percentages increased over successive generations in the two lines with introduced *tam* mutations (Figure 2C). After five generations, *mt:ATP6[1]* reached 100%. We conclude that reducing the gene dose of *tam* can increase the transmission of a distinct functional mitochondrial genome.

In the two tested heteroplasmic lines, mtDNA competition is influenced by both purifying and selfish selection, so the effect of *tam* dose could be due to enhancement of purifying selection to benefit the functional genome (*mt:yak* or *mt:ATP6[1]*) or diminution of the selfish transmission advantage of *mt:ND2*^{del1}+*CoI*^{T300I}, or both. To address this, we transferred the *mt:yak/mt:ND2*^{del1}+*CoI*^{T300I} lines to a lower temperature (22°C), where purifying selection against the *mt:ND2*^{del1}+*CoI*^{T300I} is greatly diminished because of improved function of the mutant [5]. At this temperature, selfish selection dominates the competition, and *mt:yak* percentage declined and became undetectable in two generations in control fly groups (Figure 3A; also described in [8]). When *tam* was heterozygous, *mt:yak* declined just as it did

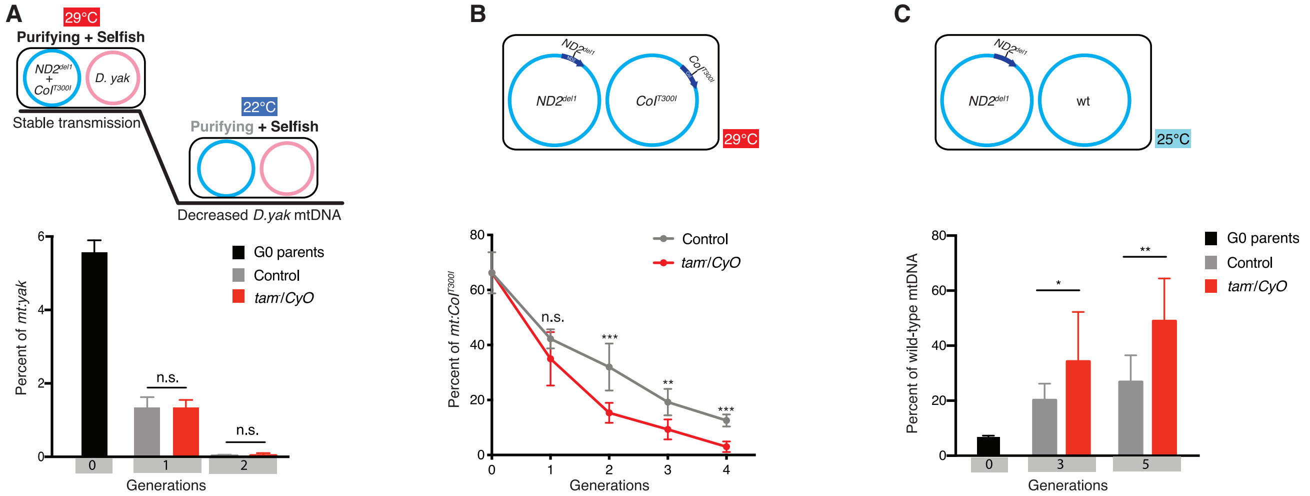

Figure 3. Reducing *tam* Gene Dose Enhances Purifying Selection

(A) *tam* heterozygosity showed no effect on the dynamics of the *mt:yak* decline when the *mt:yak/mt:ND2^{del1}+CoI^{T300I}* line was cultivated at 22°C, where the purifying selection against the *mt:ND2^{del1}+CoI^{T300I}* genome was significantly reduced. In both control and *tam* mutant backgrounds (*tam^{3}*, *tam^{4}*, or BSC252; balanced by *CyO*), *mt:yak* was eliminated in two generations.

(B) The decline of *mt:CoI^{T300I}* in the *mt:ND2^{del1}/mt:CoI^{T300I}* line at the restrictive temperature was accelerated in heterozygous *tam* mutants. The percentage of *mt:CoI^{T300I}* was followed in various flies with only one functional copy of *tam* (*tam^{3}*, *tam^{KO}*, BSC252, or BSC812; balanced by *CyO*) and *Kr^{If}/CyO* nuclear background over generations.

(C) Reducing *tam* gene dose enhanced the rate at which wild-type mtDNA overtook *mt:ND2^{del1}* at 25°C. The percentage of the wild-type mtDNA was followed in various heterozygous *tam* mutants (*tam^{3}*, *tam^{KO}*, BSC252, or BSC812; balanced by *CyO*) and *Kr^{If}/CyO* nuclear background.

Error bars indicate SDs of three or more independent experiments; p values: Student's t test. All the controls refer to *Kr^{If}/CyO*.

in control flies (Figure 3A). Thus, at least in this experimental context, the dose of *tam* has little or no effect when selfish selection has the dominant influence. Either *tam* dose does not act on selfish selection or *tam* dose is unable to act on selfish selection at this temperature.

Next, we examined the effect of *tam* dose in a heteroplasmic combination where only purifying selection affects mtDNA competition. We previously showed that when the *mt:ND2^{del1}* genome was paired with the *mt:CoI^{T300I}* genome, *mt:ND2^{del1}* steadily outcompetes *mt:CoI^{T300I}* because of a difference in the oxidative phosphorylation (OXPHOS) function at 29°C [5]. These two genomes share the same non-coding region, and differ only by mutations in *mt:ND2* and *mt:CoI*, so there is no selfish selection involved. We found that in flies where *tam* dose was reduced, the decline of *mt:CoI^{T300I}* was accelerated (Figure 3B), suggesting that reducing *tam* enhances purifying selection. To further test whether *tam* dose acts only on *mt:CoI^{T300I}* or is only manifested at 29°C, we generated another heteroplasmic line where the *mt:ND2^{del1}* mutant was paired with the wild-type mtDNA and followed the heteroplasmy dynamics at 25°C. The *mt:ND2^{del1}* allele is slightly compromised for OXPHOS function and is slowly displaced by the wild type because of a weak purifying selection [5] (Figure 3C). In lines with only one functional genomic copy of *tam*, the wild-type mtDNA took over faster (Figure 3C). Thus, reducing *tam* acts to enhance two distinct examples of purifying selection.

For all the experiments described above, genetic crosses were designed to minimize nuclear background differences and all tested deletions and mutations were heterozygous with a balancer chromosome (*CyO* for all presented data and *TM6B* for the 3rd chromosome deficiencies; see Figure 1B and STAR Methods). However, this left open the possibility that the dose effect on heteroplasmy dynamics we observed for *tam* is specific for the given nuclear background. To test whether this is the case, we altered the crossing scheme and examined the consequence of *tam* heterozygosity with different second chromosomes. We found that, when chromosomes unrelated to the *CyO* balancer were used, the dose of *tam* had only negligible effect on the *mt:yak* percentage (Figure 4A). This suggests that the *CyO* chromosome carries one or more polymorphisms that synergize with the *tam* dose to create the observed phenotype. We hypothesized that the *CyO* balancer provided less *tam* function than other chromosomes (hypomorphic for *tam*). Such behavior might be attributed to either direct changes in the *tam* sequence or other modifying mutations that reduce the functional output of Tam. We tested various balancer chromosomes with related origins but different polymorphisms in the *tam* sequence. Although heterozygosity for *tam* gave a phenotype with all of these balancers, the strength of the phenotype varied. This variation combined with sequences of *tam* from these balancers (Figure S4A) and a test of *tam* expression from *CyO* (Figure S4B) suggests that the modification is complex and might either be caused by diverse polymorphisms associated with *tam* on the balancer or unrelated modifiers.

To avoid the complicated genetic interactions with the *CyO* balancer, we tested whether a more substantial change in *tam* alone is sufficient to modulate mtDNA competition. We used CRISPR/Cas9-based mutagenesis to isolate mutations missing a single amino acid in the highly conserved exonuclease domain of *tam* (*tam$^{\Delta 262Y}$* and *tam$^{\Delta 263D}$*) (Figure S2). These alleles are homozygous viable, and also transheterozygous viable with *tam^{3}*, *tam^{4}*, and *tam^{KO}*. We found that after introducing two copies of *tam$^{\Delta 262Y}$*

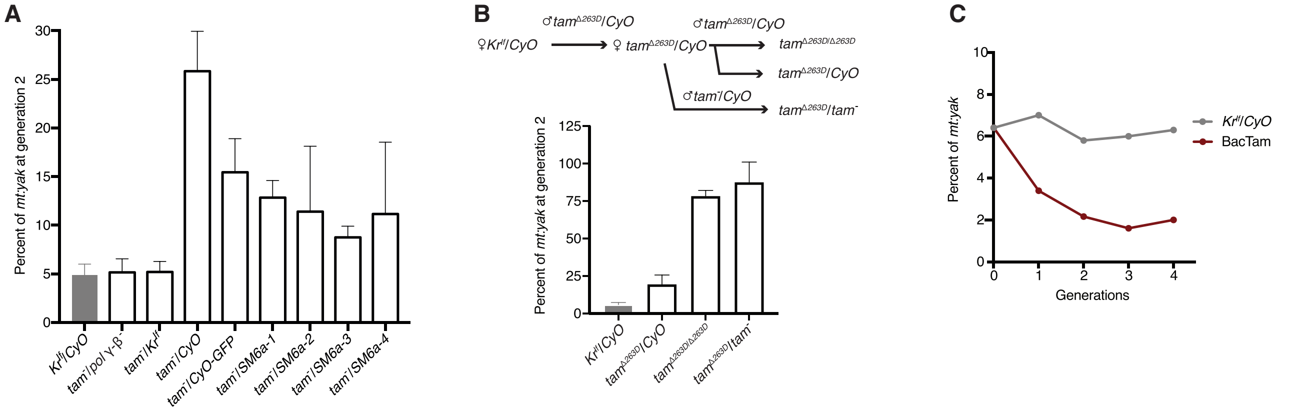

Figure 4. Modulating Tam Function Alone Is Sufficient to Influence Competition between Mitochondrial Genomes

(A) The dose effect of *tam* on the percentage of *mt:yak* was not detected in nuclear backgrounds where 2nd chromosomes unrelated to the *CyO* balancer were used. The *mt:yak* percentage was followed in various heterozygous *tam* mutants (tam^3, tam^{KO}, BSC252, or BSC812) balanced by *CyO*-related or unrelated 2nd chromosomes. A detailed cross scheme for each genotype is presented in Figure S4D. Error bars indicate SDs of three or more independent experiments.

(B) Homozygous or transheterozygous viable *tam* mutants showed that reducing functional Tam alone is sufficient to increase the percentage of *mt:yak* (see also Figure S4). Top: the cross scheme used to introduce various *tam* alleles into the stable heteroplasmic line. The *mt:yak* percentage was followed in $tam^{\Delta 263D}/CyO$, $tam^{\Delta 263D}/tam^{\Delta 263D}$, and $tam^{\Delta 263D}/tam^-$ (tam^3 *or* tam^{KO}) adult males (see Figure S2 for the sequence details of $tam^{\Delta 263D}$). Error bars indicate SDs of three or more independent experiments.

(C) Increasing *tam* dose decreased the percentage of *mt:yak*. The stable heteroplasmic females were crossed to males homozygous for *tam-GFP* on the 3rd chromosome (BAC clone; a gift from Hong Xu, NIH) for two generations to produce heteroplasmic flies containing two endogenous copies of *tam* on the 2nd chromosome and two extra copies of *tam-GFP* on the 3rd chromosome. The *mt:yak* percentage was followed for four generations.

or $tam^{\Delta 263D}$ in the $mt{:}yak/mt{:}ND2^{del1}+CoI^{T300I}$ line, the *mt:yak* percentage increased to over 75% at generation 2. Similarly, the *mt:yak* percentage in transheterozygous $tam^-/tam^{\Delta 262Y}$ or $tam^-/tam^{\Delta 263D}$ increased to over 70% at generation 2 (Figures 4B and S4C). These data suggest that compromising *tam* function alone is sufficient to modify the competition between mitochondrial genomes, but Tam function probably needs to be reduced to less than 50%. Additionally, we analyzed the consequence of an increase in *tam* dose by following flies with two extra copies of *tam* (BacTam). Over a few generations at 29°C, the percentage of *mt:yak* fell from 6% to 2% in the BacTam background (Figure 4C). All the above data suggest that altering *tam* functional output alone is sufficient to influence the transmission of detrimental mitochondrial genomes.

Our findings show that the balance that maintains a stable heteroplasmic state is precarious and modified by many genetic loci and that the shift in function of one of these, *tam*, can drive the elimination of a detrimental mitochondrial mutation that was otherwise stably inherited for many generations. The discovery that the gene involved encodes the mtDNA polymerase suggests a connection with replication of the genomes, but the genetic analysis reported here does not directly reveal the mechanism. It is tempting to speculate that reducing Tam activity might favor the replication of the diverged *mt:yak* genome, but no such favoritism was observed when the temperature was reduced to minimize the functional difference between *mt:yak* and the $mt{:}ND2^{del1}+CoI^{T300I}$ genome. Furthermore, at 25°C, the lower dose of *tam* still favored the functional genome when two *D. melanogaster* mitochondrial genomes were pitted against each other (Figure 3C). This suggests that the differential effect of *tam* dose impacts the effectiveness of purifying selection. Although speculative, such an effect might be explained by the involvement of Tam in a newly advanced quality control mechanism. Recently, Zhang et al. showed that PINK1, a kinase sensitive to mitochondrial potential, is selectively stabilized on the surface of mitochondria enriched for mutant genomes [24]. They further showed that PINK1 phosphorylates Larp to inhibit local translation of nuclear-encoded mitochondrial proteins on the surface of the unfit mitochondria. Tam was one of the factors whose expression was dramatically reduced by this signaling pathway. They proposed that reduced translation starves unfit mitochondria of nuclear-encoded replication factors. Accordingly, Tam could be a key factor limiting replication in unfit mitochondria when it falls below a certain threshold. A reduction in the dose could promote the action of this system by making it easier to reach the threshold that starves the unfit mitochondria of this limiting factor. However, cell biological and disease phenotypes of *POLG* mutations are diverse, suggesting the existence of alternative possible explanations for how dose change can impact the balance of heteroplasmic genomes [25–28].

Regardless of the mechanism, our genetic findings reveal numerous nuclear loci that affect the competition between mitochondrial genomes, suggesting that multiple pathways influence the selective forces defining the outcome of competition. Perhaps reflecting complex inputs, the magnitude of the impact of *tam* gene dose differs strikingly in different genetic backgrounds. In the two examples where diverged mitochondrial genomes are differently favored by selfish and purifying selection, reduction of *tam* dose completely reverses the outcome of the competition such that the winner becomes the loser and is eliminated (Figures 2B and 2C). This outcome seems out of proportion with subtler shifts in the strength of purifying selection assessed in other heteroplasmic backgrounds (Figures 3B and 3C). Perhaps as-yet unappreciated differences in the interaction of Tam with the much altered regulatory regions of these competing genomes increase the sensitivity to *tam* dose in these competitions.

In conclusion, the genetic approach we have used here has the potential of defining in some detail the largely unknown rules of nuclear management of mtDNA transmission (e.g., [14–18]). The pervasive impact of a change in the level of mtDNA polymerase catalytic subunit shows the potency of the nuclear influence on the success of mitochondrial genomes, a factor that would affect the heritance of heteroplasmic mitochondrial disease traits. The large number of loci that influence selection suggests that nuclear management of mitochondrial evolution is deeply entrenched. We propose that it has played a role throughout eukaryotic evolution in taming and subjugating the genome of an infecting microbe to adopt its current role. Because many mitochondrial diseases are carried in heteroplasmy, the extensive nuclear inputs might identify pharmacologically accessible pathways whose manipulation could provide clinical benefit.

STAR★METHODS

Detailed methods are provided in the online version of this paper and include the following:

- KEY RESOURCES TABLE
- LEAD CONTACT AND MATERIALS AVAILABILITY
- EXPERIMENTAL MODEL AND SUBJECT DETAILS
- METHOD DETAILS
  - The deficiency screen cross scheme
  - CRISPR/Cas9-based mutagenesis
  - DNA extraction and quantitative PCR
  - Total RNA extraction and reverse transcription
  - Embryo mtDNA extraction and copy number measurement
- QUANTIFICATION AND STATISTICAL ANALYSIS
- DATA AND CODE AVAILABILITY

SUPPLEMENTAL INFORMATION

Supplemental Information can be found online at https://doi.org/10.1016/j.cub.2019.10.060.

ACKNOWLEDGMENTS

We would like to thank Dr. Hong Xu (National Heart, Lung, and Blood Institute) for generously sharing the BacTam flies and Prof. Nils-Goran Larsson (Karolinska Institutet) for sharing the *tam*^KO mutant. This work is funded by NIH grant GM120005 to P.H.O. and Wellcome Trust grant 202269/Z/16/Z to H.M.

AUTHOR CONTRIBUTIONS

Conceptualization, A.C.-Y.C., H.M., and P.H.O.; Investigation, A.C.-Y.C., E.M., and H.M.; Methodology, A.C.-Y.C. and H.M.; Writing – Original Draft, P.H.O. and H.M.; Writing – Review & Editing, P.H.O. and H.M.; Funding Acquisition, P.H.O. and H.M.; Supervision, P.H.O. and H.M.

DECLARATION OF INTERESTS

The authors declare no competing interests.

Received: April 18, 2019
Revised: October 9, 2019
Accepted: October 29, 2019
Published: November 27, 2019

REFERENCES

1. Stewart, J.B., Freyer, C., Elson, J.L., Wredenberg, A., Cansu, Z., Trifunovic, A., and Larsson, N.G. (2008). Strong purifying selection in transmission of mammalian mitochondrial DNA. PLoS Biol. *6*, e10–e71.
2. Freyer, C., Cree, L.M., Mourier, A., Stewart, J.B., Koolmeister, C., Milenkovic, D., Wai, T., Floros, V.I., Hagström, E., Chatzidaki, E.E., et al. (2012). Variation in germline mtDNA heteroplasmy is determined prenatally but modified during subsequent transmission. Nat. Genet. *44*, 1282–1285.
3. Fan, W., Waymire, K.G., Narula, N., Li, P., Rocher, C., Coskun, P.E., Vannan, M.A., Narula, J., Macgregor, G.R., and Wallace, D.C. (2008). A mouse model of mitochondrial disease reveals germline selection against severe mtDNA mutations. Science *319*, 958–962.
4. Floros, V.I., Pyle, A., Dietmann, S., Wei, W., Tang, W.C.W., Irie, N., Payne, B., Capalbo, A., Noli, L., Coxhead, J., et al. (2018). Segregation of mitochondrial DNA heteroplasmy through a developmental genetic bottleneck in human embryos. Nat. Cell Biol. *20*, 144–151.
5. Ma, H., Xu, H., and O'Farrell, P.H. (2014). Transmission of mitochondrial mutations and action of purifying selection in *Drosophila melanogaster*. Nat. Genet. *46*, 393–397.
6. Hill, J.H., Chen, Z., and Xu, H. (2014). Selective propagation of functional mitochondrial DNA during oogenesis restricts the transmission of a deleterious mitochondrial variant. Nat. Genet. *46*, 389–392.
7. Lieber, T., Jeedigunta, S.P., Palozzi, J.M., Lehmann, R., and Hurd, T.R. (2019). Mitochondrial fragmentation drives selective removal of deleterious mtDNA in the germline. Nature *570*, 380–384.
8. Ma, H., and O'Farrell, P.H. (2016). Selfish drive can trump function when animal mitochondrial genomes compete. Nat. Genet. *48*, 798–802.
9. Tsang, W.Y., and Lemire, B.D. (2002). Stable heteroplasmy but differential inheritance of a large mitochondrial DNA deletion in nematodes. Biochem. Cell Biol. *80*, 645–654.
10. Volz-Lingenhöhl, A., Solignac, M., and Sperlich, D. (1992). Stable heteroplasmy for a large-scale deletion in the coding region of *Drosophila subobscura* mitochondrial DNA. Proc. Natl. Acad. Sci. USA *89*, 11528–11532.
11. Clark, K.A., Howe, D.K., Gafner, K., Kusuma, D., Ping, S., Estes, S., and Denver, D.R. (2012). Selfish little circles: transmission bias and evolution of large deletion-bearing mitochondrial DNA in *Caenorhabditis briggsae* nematodes. PLoS ONE *7*, e41433.
12. Phillips, W.S., Coleman-Hulbert, A.L., Weiss, E.S., Howe, D.K., Ping, S., Wernick, R.I., Estes, S., and Denver, D.R. (2015). Selfish mitochondrial DNA proliferates and diversifies in small, but not large, experimental populations of *Caenorhabditis briggsae*. Genome Biol. Evol. *7*, 2023–2037.
13. Kang, E., Wu, J., Gutierrez, N.M., Koski, A., Tippner-Hedges, R., Agaronyan, K., Platero-Luengo, A., Martinez-Redondo, P., Ma, H., Lee, Y., et al. (2016). Mitochondrial replacement in human oocytes carrying pathogenic mitochondrial DNA mutations. Nature *540*, 270–275.
14. Battersby, B.J., Loredo-Osti, J.C., and Shoubridge, E.A. (2003). Nuclear genetic control of mitochondrial DNA segregation. Nat. Genet. *33*, 183–186.
15. Matsuura, E.T., Tanaka, Y.T., and Yamamoto, N. (1997). Effects of the nuclear genome on selective transmission of mitochondrial DNA in *Drosophila*. Genes Genet. Syst. *72*, 119–123.
16. Farge, G., Touraille, S., Le Goff, S., Petit, N., Renoux, M., Morel, F., and Alziari, S. (2002). The nuclear genome is involved in heteroplasmy control in a mitochondrial mutant strain of *Drosophila subobscura*. Eur. J. Biochem. *269*, 998–1005.
17. Jenuth, J.P., Peterson, A.C., and Shoubridge, E.A. (1997). Tissue-specific selection for different mtDNA genotypes in heteroplasmic mice. Nat. Genet. *16*, 93–95.
18. Jokinen, R., Marttinen, P., Sandell, H.K., Manninen, T., Teerenhovi, H., Wai, T., Teoli, D., Loredo-Osti, J.C., Shoubridge, E.A., and Battersby, B.J. (2010). Gimap3 regulates tissue-specific mitochondrial DNA segregation. PLoS Genet. *6*, e1001161.

19. Roote, J., and Russell, S. (2012). Toward a complete *Drosophila* deficiency kit. Genome Biol. *13*, 149.
20. Cook, R.K., Christensen, S.J., Deal, J.A., Coburn, R.A., Deal, M.E., Gresens, J.M., Kaufman, T.C., and Cook, K.R. (2012). The generation of chromosomal deletions to provide extensive coverage and subdivision of the *Drosophila melanogaster* genome. Genome Biol. *13*, R21.
21. Iyengar, B., Roote, J., and Campos, A.R. (1999). The tamas gene, identified as a mutation that disrupts larval behavior in *Drosophila melanogaster*, codes for the mitochondrial DNA polymerase catalytic subunit (DNApol-gamma125). Genetics *153*, 1809–1824.
22. Bratic, A., Kauppila, T.E.S., Macao, B., Grönke, S., Siibak, T., Stewart, J.B., Baggio, F., Dols, J., Partridge, L., Falkenberg, M., et al. (2015). Complementation between polymerase- and exonuclease-deficient mitochondrial DNA polymerase mutants in genomically engineered flies. Nat. Commun. *6*, 8808.
23. Ma, H., and O'Farrell, P.H. (2015). Selections that isolate recombinant mitochondrial genomes in animals. eLife *4*, e07247.
24. Zhang, Y., Wang, Z.-H., Liu, Y., Chen, Y., Sun, N., Gucek, M., Zhang, F., and Xu, H. (2019). PINK1 inhibits local protein synthesis to limit transmission of deleterious mitochondrial DNA mutations. Mol. Cell *73*, 1127–1137.e5.
25. Yu, Z., O'Farrell, P.H., Yakubovich, N., and DeLuca, S.Z. (2017). The mitochondrial DNA polymerase promotes elimination of paternal mitochondrial genomes. Curr. Biol. *27*, 1033–1039.
26. Peeva, V., Blei, D., Trombly, G., Corsi, S., Szukszto, M.J., Rebelo-Guiomar, P., Gammage, P.A., Kudin, A.P., Becker, C., Altmüller, J., et al. (2018). Linear mitochondrial DNA is rapidly degraded by components of the replication machinery. Nat. Commun. *9*, 1727.
27. Nissanka, N., Bacman, S.R., Plastini, M.J., and Moraes, C.T. (2018). The mitochondrial DNA polymerase gamma degrades linear DNA fragments precluding the formation of deletions. Nat. Commun. *9*, 2491.
28. Chan, S.S.L., and Copeland, W.C. (2009). DNA polymerase gamma and mitochondrial disease: understanding the consequence of POLG mutations. Biochim. Biophys. Acta *1787*, 312–319.

STAR★METHODS

KEY RESOURCES TABLE

REAGENT or RESOURCE	SOURCE	IDENTIFIER
Experimental Models: Organisms/Strains		
D. melanogaster: Bloomington Deficiency Kit	Bloomington *Drosophila* Stock Center	https://bdsc.indiana.edu/stocks/df/dfkit.html
D. melanogaster: *twk* mutants (*twk*^{1} and *twk*^{2}), *GatC* mutants (*GatC*^{1} and *GatC*^{2}), *tam* mutants (*tam*$^{\Delta 262Y}$ and *tam*$^{\Delta 263D}$), *pol* γ-β mutants (*pol* γ-β^{1} and *pol* γ-β^{2}), see Figure S2	This paper	N/A
D. melanogaster: tam^{KO}	[22]	N/A
D. melanogaster: BacTam	Hong Xu (NIH)	N/A
D. melanogaster: *tam*^{3}	Bloomington *Drosophila* Stock Center	BDSC:3410
D. melanogaster: *tam*^{4}	Bloomington *Drosophila* Stock Center	BDSC:25145
D. melanogaster: Df(2L)Exel7059,	Bloomington *Drosophila* Stock Center	BDSC:7826
D. melanogaster: Df(2L)FDD-0428643	Bloomington *Drosophila* Stock Center	BDSC:25166
D. melanogaster: Df(2L)Exel7043	Bloomington *Drosophila* Stock Center	BDSC:7816
D. melanogaster: *nos-Cas9*	Bloomington *Drosophila* Stock Center	BDSC:54591
Oligonucleotides		
Primers for qPCR, see Table S4	This paper	N/A
Primers for RT-qPCR, see Table S4	This paper	N/A
Recombinant DNA		
pCDF5+guide RNA for CRISPR/Cas9-based editing for the following genes (*twk*, *GatC*, *tam* and *pol* γ-β)	This paper	N/A
Software and Algorithms		
Guide RNA design for the following genes (*twk*, *GatC*, *tam* and *pol* γ-β)	FLYCRISPR	https://flycrispr.org/
Other		
Sequence data of *tam* ROF of a number of 2nd chromosomes (*D. melanogaster*), See Figure S4A	This paper	N/A

LEAD CONTACT AND MATERIALS AVAILABILITY

Further information and requests for resources and reagents should be directed to and will be fulfilled by the Lead Contact, Hansong Ma (hm555@cam.ac.uk). There are no restrictions to the availability of reagents.

EXPERIMENTAL MODEL AND SUBJECT DETAILS

The following flies were used in this study: Bloomington Deficiency Kits (Table S1), additional deficiency lines that cover the entire *tam* gene (Df(2L)Exel7059 and Df(2L)FDD-0428643) or *twk* gene (Df(2L)Exel7043) (BDSC:7826, BDSC:21566 and BDSC:7816, respectively), *tam*^{3} and *tam*^{4} (BDSC:3410 and BDSC:25145), *tam*^{KO} (generated in [22]), BacTam (a gift from Hong Xu, National Heart, Lung and Blood Institute), *nos-Cas9* (BDSC:54591) and four lines carrying the *SM6a* balancer chromosome. All fly stocks were raised on standard media at 25°C unless otherwise stated.

Various heteroplasmic lines were generated via cytoplasmic transplantation as described in [5]. The stable *mt:yak/mt:ND2*^{del1}+*CoI*^{T300I} was previously created by introducing cytoplasm of *D. yakuba* into the *mt:ND2*^{del1}+*mt:CoI*^{T300I} embryos [8]. After the stable heteroplasmy was established, flies were balanced on the 2nd or 3rd chromosome by crossing to *Kr*^{If}*/CyO* or *MKRS/TM6B* males, respectively. Once balanced, the flies were continuously backcrossed to *Kr*^{If}*/CyO* or *MKRS/TM6B* males to maintain an isogenic nuclear background. To create *mt:ATP6[1]/mt:ND2*^{del1}+*CoI*^{T300I} line, cytoplasm of *mt:ND2*^{del1}+*CoI*^{T300I} was injected into *mt:ATP6[1]* embryos in order to create founder flies with a high percentage of *mt:ATP6[1]*. To generate *mt:ND2*^{del1}*/mt:CoI*^{T300I}*,* cytoplasm of *mt:ND2*^{del1} embryos was transferred to *mt:CoI*^{T300I} embryos to create heteroplasmic flies with a high percentage of *mt:CoI*^{T300I}. To generate wild-type/*mt:ND2*^{del1}*,* cytoplasm of wild-type embryos was transferred to *mt:ND2*^{del1} embryos to create heteroplasmic flies with a high percentage of *mt:ND2*^{del1}. All the heteroplasmic lines were maintained and examined at 29°C, except the wild-type/*mt:ND2*^{del1} flies, which were maintained at 25°C instead.

METHOD DETAILS

The deficiency screen cross scheme

The screen was carried out at 29°C as shown in Figure 1B. Basically, for each deficiency, 5 males carrying the deletion chromosome were mated with 10 heteroplasmic females (generation 0) balanced with either Kr^{If}/*CyO* (for 2nd chromosome deficiencies) or *MKRS/TM6B* (for 3rd chromosome deficiencies). After one generation, more than 10 female progeny (generation 1) with an individual deletion chromosome balanced by *CyO or TM6B* were mated with 10 Kr^{If}/*CyO* or *MKRS/TM6B* males to maintain the deficiency and minimize variations in the nuclear background. Total DNA from 10 to 40 young male progeny (generation 2) that carry the deletion chromosome (balanced by *CyO* or *TM6B*) was extracted for qPCR analysis. Heterozygous mutants of *tam*, *pol* γ-β, *GatC*, and *twk* were tested with the same experimental setup for every generation. For controls, Kr^{If}/*CyO* males were used instead of deficiency males for the first cross.

CRISPR/Cas9-based mutagenesis

CRISPR/Cas9-based mutagenesis was performed as described on FlyCRISRP (https://flycrispr.org/). In brief, two gRNAs were designed for each of the following genes: *pol* γ-β (gaaaaacgctggatgttgac, gctttgatgtttcagaagag), *GatC* (gcagctaacgcatcccacca, gatctggatttcggaggcgc), *twk* (tgctggcttacgtaaacaag, atatctgggcgatcgacggg), or *tam* (gtcacaatgtctcctacgac, ctacgacagggcgcgac tga) using FlyCRISPR target finder (http://tools.flycrispr.molbio.wisc.edu/targetFinder/). Complimentary oligos were synthesized by Integrated DNA Technologies and were cloned into a pCFD5 plasmid. Plasmids were amplified and purified, and then injected into *nos-Cas9* flies (BDSC:54591) at a concentration of 200 ng/μl. Adults were then balanced by crossing to Kr^{If}/*CyO* twice to establish individual stocks. The mutated sequences were verified by Sanger sequencing.

DNA extraction and quantitative PCR

Total DNA extraction was performed as described in [5]. In brief, 10 to 40 adult males were squashed in 500 μL of homogenization buffer (100 mM Tris-HCl (pH 8.8), 10 mM EDTA, 1% SDS) and incubated at 70°C for 30 min. Potassium acetate was added to a final concentration of 1 M, and samples were incubated on ice for 30 min. Samples were centrifuged at 20,000 g for 10 min at room temperature. DNA was recovered from the supernatant by adding 0.5x volume of isopropanol followed by washing with 70% ethanol. DNA was then dissolved in 100 μL Tris (10 mM, pH 8.0) before further dilution.

For all qPCR reactions, 2X SensiFast SYBR Green PCR Master Mix (Bioline 98020) was used in 20 μL reactions with 500 nM of each primer. For each reaction, 5% of a male's total genomic DNA was used as the template to allow the Ct values to land between cycles 10-25. Each qPCR cycle was incubated at 95°C for 10 min followed by 35 cycles of 95°C for 30 s and 48°C for 30 s. Standard curves were plotted using a series of tenfold dilutions (2×10^7 to 2×10^3 copies per qPCR reaction) of the linearized PCR products containing regions covered by both the common and specific primer sets. The efficiency of each primer set was normalized by comparison to homoplasmic mtDNA that contain both the common and specific region. The absolute copy number of targeted regions was calculated according to the Ct value and the standard curve for one of the co-resident mtDNA genotypes (e.g., *mt:yak*, recognized by the specific primer set) and total mtDNA (recognized by the common primer set). All the primers are listed in Table S4.

Total RNA extraction and reverse transcription

Total RNA from 2-day old males was extracted based on the TRIzol reagent (Invitrogen) protocol. Ten males were ground with 750 μL of TRIzol reagent and incubated at room temperature for 10 min. Phenol was removed from samples by multiple rounds of chloroform extraction. RNA from the supernatant was precipitated by adding 0.5x isopropanol and washed once with 70% ethanol. The extracted RNA was then treated with RNase-free DNase I (New England Biolabs) for 30 min at 37°C to remove genomic DNA. Subsequently, DNase activity was heat-inactivated for 10 min at 65°C upon adding 1 μL of 50 mM EDTA. The RNA was then reverse-transcribed with Oligo $(dT)_{18}$ primer using RevertAid First-strand cDNA synthesis kit (Invitrogen). The relative expression level of *tam* and *pol* γ-β was measured by qPCR and normalized to the expression level of house-keeping gene *Act42A* or *EF1α*. For each qPCR reaction, 2X SensiFast SYBR Green PCR Master mix (Bioline) was used in 20 μL reactions with 500 nM of each primer. The qPCR cycle was set as 95°C for 10 min followed by 35 cycles of 95°C for 30 s and elongation for 30 s. All the primers are listed in Table S4.

Embryo mtDNA extraction and copy number measurement

For each genotype, over 50 newly laid eggs (collected within 20 min after egg laying) were lysed in 100 μL of QuickExtract buffer (Lucigen, Thermo Fisher Scientific) in Precellys homogenizer. In brief, samples were agitated 3 times at 4,000 rpm for 30 s with a 30 s pause at room temperature. The homogenized samples were then incubated for 15 min at 65°C followed by 5 min at 95°C. The total mtDNA copy number was then measured by qPCR using the common primer set that binds to a conserved mtDNA region of *mt:yak* and $mt{:}ND2^{del1}{+}CoI^{T300I}$ (Table S4).

QUANTIFICATION AND STATISTICAL ANALYSIS

As specified in all figure legends, the percentage of a given mitochondrial genotype in a heteroplasmic line (Figures 1C, 2A, 3, 4A, 4B, and S3C) was measured in at least three independent biological replicates. Each replicate contained 10-40 young adult males.

Similarly, the total mtDNA copy number (Figure S3B) and the mRNA level of *tam* and *pol* γ-β genes (Figures S3A and S4B) were measured in three independent biological replicates. For mtDNA copy number quantifications, 50 newly laid eggs or ten 2-day old adult males were used for each replicate. For mRNA level quantifications, each replicate included ten 2-day old adult males. Figures are all presented as mean ± SD. All the statistical analyses were performed using GraphPad Prism 7.0. Differences were examined by unpaired Student's t test. Significance was defined by *p < 0.05, **p < 0.005, and ***p < 0.0005.

DATA AND CODE AVAILABILITY

This study did not generate datasets and codes.

Current Biology, Volume *29*

Supplemental Information

A Genome-wide Screen Reveals that Reducing Mitochondrial DNA Polymerase Can Promote Elimination of Deleterious Mitochondrial Mutations

Ason C.-Y. Chiang, Eleanor McCartney, Patrick H. O'Farrell, and Hansong Ma

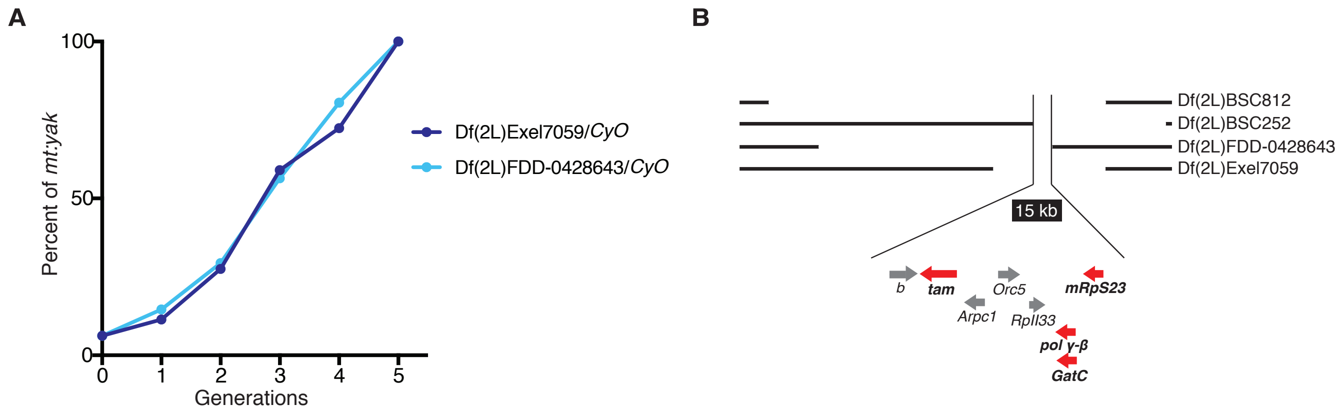

Figure S1. Two additional deficiencies that cover the *tam* genomic region showed an increased *mt:yak* percentage over generations. Related to Figure 1.

A) In Df(2L)Exel7059 and Df(2L)FDD-0428643 lines, the *mt:yak* took over after five generations. **B)** The common region deleted by the four deficiencies that similarly modified the heteroplasmy dynamic is indicated. It is 15 kb in size and contains eight genes, four of which encode mitochondrial proteins (red).

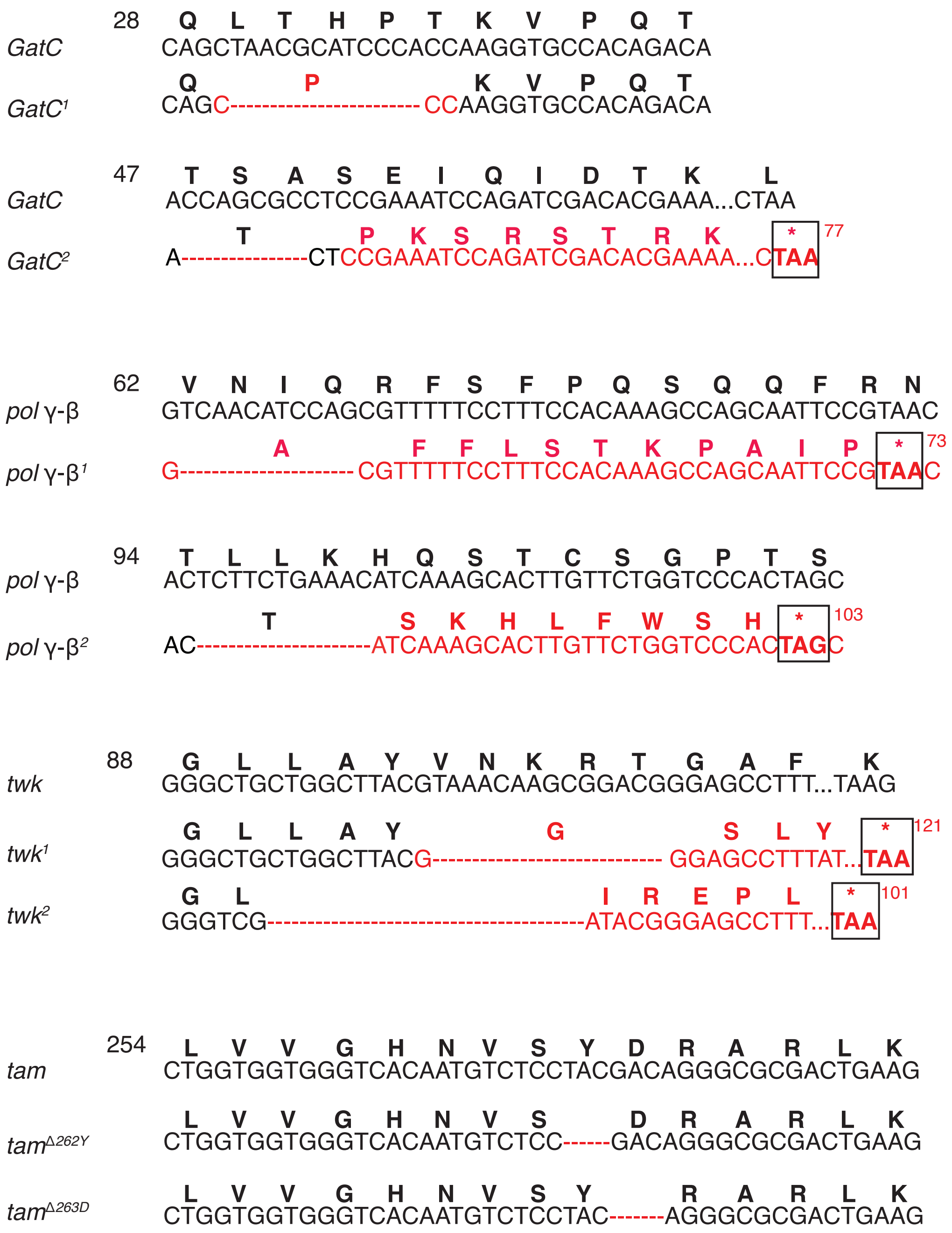

Figure S2. Sequence details of *GatC*, *pol* γ-β, *twk* and *tam* mutants generated by CRISPR/Cas9-based editing. Related to Figures 2, 4, S3 and S4.

Wild-type DNA and amino acid sequences (bold) are displayed in parallel to the mutant sequences with deletions (hyphen lines) that lead to frameshift changes in amino acid sequence (red) and a stop codon at the position indicated (asterisk, number of amino acid residues incorporated). The lethal phase of homozygote *twk* and *pol* γ-β mutants was in early pupation, whereas homozygous *GatC* mutants only reach late embryonic or early 1st instar larval stages. The *GatC* mutants were trans-heterozygous viable when crossed to the two *pol* γ-β mutants, indicating that the isolated mutants affected the targeted gene without inactivating its co-transcribed partner. Each *tam* mutant carries a 3 bp deletion, which removes a single amino acid at the highly conserved exonuclease domain. Both are homozygous viable but female sterile. For the amino acid residue 263, a D to A mutation has been previously reported to increase mtDNA mutation load over generations in *Drosophila* [S1,S2].

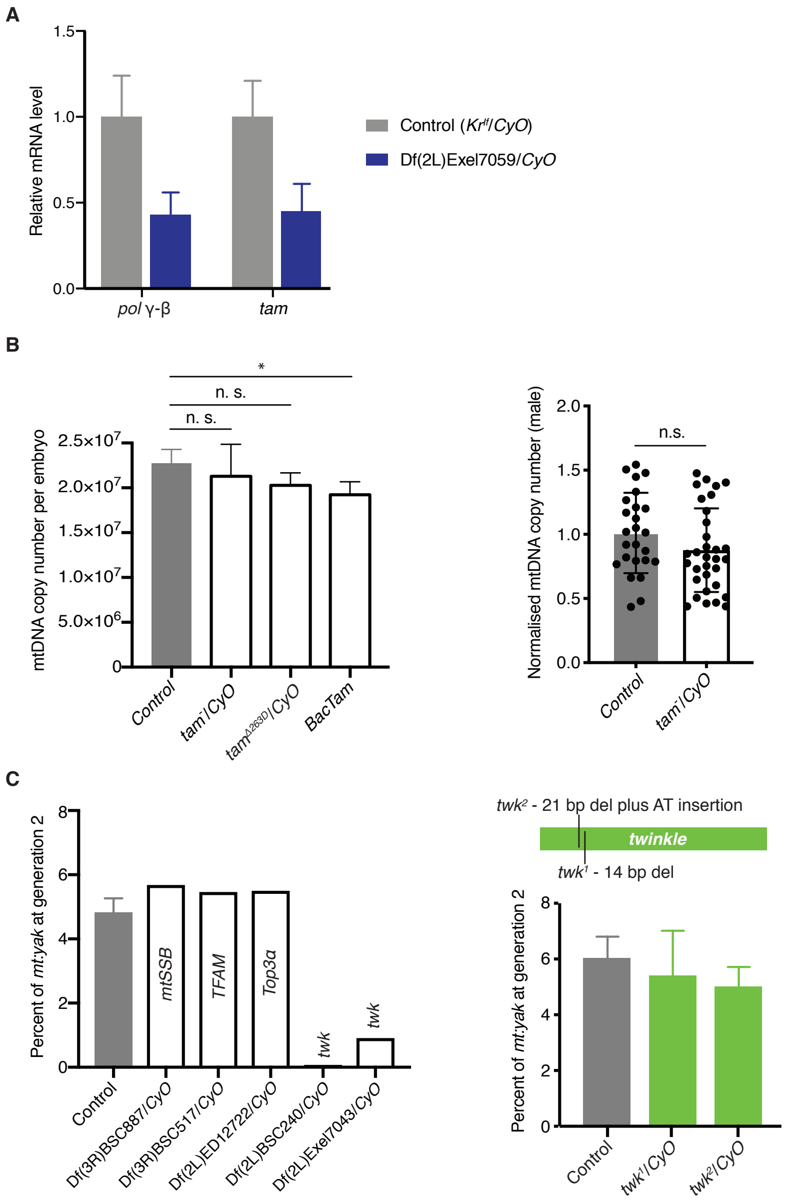

Figure S3. Characterisation of different deficiencies and heterozygous mutants to assess the impact of replication proteins on the total mtDNA copy number and mtDNA competition. Related to Figure 2.

A) The relative mRNA levels of *tam* and *pol γ-β* in adult male of Df(2L)Exel7059 were about half compared to those of Kr^{If}/*CyO*. Each sample represents an independent RNA extraction from a group of ten 2-day old adult males (n=3, Student's *t*-test, error bars: SDs). **B)** The heterozygous *tam* mutants (tam^{3}, tam^{4}, tam^{KO} and $tam^{\Delta 263D}$) have a similar total mtDNA copy number compared to Kr^{If}/*CyO* flies. Left panel: the absolute mtDNA copy number per egg was measured in newly laid eggs by qPCR (n=3, *p*-value: Student's *t*-test, error bars: SDs). Each sample represents an extraction from a group of more than 50 eggs. Right panel: The mtDNA copy number per adult male was measured by qPCR and then normalized to the input amount of DNA. Each sample represents an extraction from a group of ten 2-day old adult males (n>25, *p*-value: Student's *t*-test, error bars: SDs). **C)** Other components of mtDNA replication machinery show no dosage-dependent impact on mtDNA competition. Left panel: the abundance of *mt:yak* was not changed in deficiency lines that delete one genomic copy of *mtSSB*, *TFAM* or *Top3α*, but was reduced in deficiencies removing one copy of *twk*. Right panel: heterozygous *twk* mutants showed no change in the *mt:yak* percentage in two generations. The two *twk* mutants were isolated by CRISPR/Cas9-based editing (Figure S2, error bars: SDs).

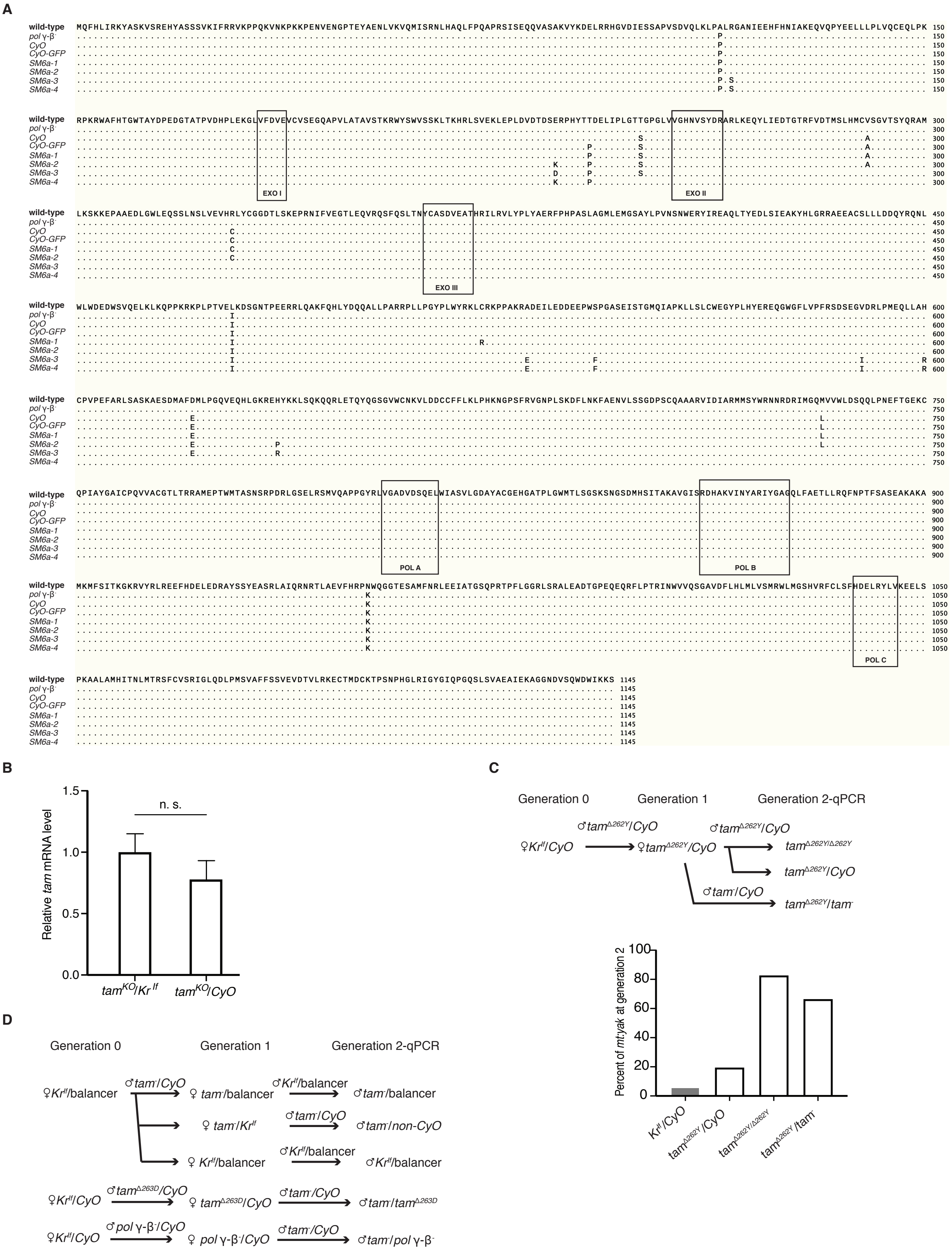
A
wild-type
pol γ-β-
CyO
CyO-GFP
SM6a-1
SM6a-2
SM6a-3
SM6a-4
EXO I
EXO II
EXO III
POL A
POL B
POL C
B
Relative tam mRNA level
n. s.
tamKO/Kr If
tamKO/CyO
C
Generation 0
Generation 1
Generation 2-qPCR
♀Krif/CyO
♂tamΔ262Y/CyO
♀tamΔ262Y/CyO
tamΔ262Y/Δ262Y
tamΔ262Y/CyO
♂tam-/CyO
tamΔ262Y/tam-
Percent of mt:yak at generation 2
Krif/CyO
D
Generation 0
Generation 1
Generation 2-qPCR
♀Krif/balancer
♂tam-/CyO
♀ tam-/balancer
♂Krif/balancer
♂tam-/balancer
♀ tam-/Krif
♂tam-/non-CyO
♀ Krif/balancer
♂Krif/balancer
♀Krif/CyO
♂tamΔ263D/CyO
♀ tamΔ263D/CyO
♂tam-/tamΔ263D
♂pol γ-β-/CyO
♀ pol γ-β-/CyO
♂tam-/pol γ-β-

Figure S4. Modulating Tam function alone is sufficient to influence competition between mitochondrial genomes. Related to Figure 4.

A) The *tam* sequence for *CyO* related or unrelated 2nd chromosomes. The detailed lineages of the *CyO* related chromosomes are uncertain and the sequences appear to be at odds with a shared heritage among *SM6a* chromosomes. Flies carrying *SM6a-2*, *SM6a-3* and *SM6a-4* were obtained from the Bloomington *Drosophila* Stock Centre, and their stock numbers are 25166, 8045 and 27383, respectively. The amino acids that do not differ from the wild-type allele are indicated by dots. The three conserved exonuclease and polymerase domains are labelled (refer to [S3]). **B)** The relative mRNA level of *tam* in tam^{KO} heterozygous with Kr^{If} or *CyO* balancer chromosomes. Each sample represents an independent RNA extraction from a group of ten 2-day old adult males (n=3, Student's *t*-test, error bars: SDs). **C)** Homozygous or transheterozygous viable *tam* mutants revealed that reducing functional Tam alone is sufficient to increase the *mt:yak* percentage. The top panel illustrates the cross scheme used to introduce various *tam* alleles into the stable heteroplasmic line. The *mt:yak* percentage was measured in $tam^{\Delta 262Y}/CyO$, $tam^{\Delta 262Y}/tam^{\Delta 262Y}$ and $tam^{\Delta 262Y}/tam^{-}$ (tam^{KO}) adult males. **D)** The cross scheme to generate tam^{-} heterozygotes with *CyO* related or unrelated 2nd chromosomes used in Figure 4A.

Table S1. A list of all deficiencies tested in this study. Related to Figure 1.

Df(2L)al	Df(2L)ED441	Df(2R)BSC702	Df(3L)BSC419	Df(3R)BSC633
Df(2L)ast2	Df(2L)ED4651	Df(2R)BSC769	Df(3L)BSC449	Df(3R)BSC650
Df(2L)BSC106	Df(2L)ED489	Df(2R)BSC780	Df(3L)BSC671	Df(3R)BSC677
Df(2L)BSC107	Df(2L)ED50001	Df(2R)BSC782	Df(3L)BSC673	Df(3R)BSC681
Df(2L)BSC109	Df(2L)ED5878	Df(2R)BSC784	Df(3L)BSC730	Df(3R)BSC728
Df(2L)BSC110	Df(2L)ED629	Df(2R)BSC787	Df(3L)BSC774	Df(3R)BSC738
Df(2L)BSC111	Df(2L)ED678	Df(2R)BSC821	Df(3L)BSC775	Df(3R)BSC741
Df(2L)BSC142	Df(2L)ED690	Df(2R)BSC865	Df(3L)BSC797	Df(3R)BSC748
Df(2L)BSC143	Df(2L)ED761	Df(2R)BSC880	Df(3L)BSC800	Df(3R)BSC749
Df(2L)BSC145	Df(2L)ED775	Df(2R)BSC883	Df(3L)BSC815	Df(3R)BSC750
Df(2L)BSC148	Df(2L)ED7853	Df(2R)BSC885	Df(3L)BSC816	Df(3R)BSC790
Df(2L)BSC149	Df(2L)ED793	Df(2R)BSC889	Df(3L)BSC845	Df(3R)BSC793
Df(2L)BSC151	Df(2L)ED8142	Df(2R)CX1	Df(3L)BSC884	Df(3R)BSC819
Df(2L)BSC159	Df(2L)ED94	Df(2R)ED1612	Df(3L)ED201	Df(3R)BSC874
Df(2L)BSC165	Df(2L)Exel6005	Df(2R)ED1673	Df(3L)ED208	Df(3R)BSC887
Df(2L)BSC169	Df(2L)Exel6009	Df(2R)ED1715	Df(3L)ED210	Df(3R)ED10639
Df(2L)BSC17	Df(2L)Exel6011	Df(2R)ED1725	Df(3L)ED217	Df(3R)ED10642
Df(2L)BSC172	Df(2L)Exel6012	Df(2R)ED1742	Df(3L)ED229	Df(3R)ED10845
Df(2L)BSC180	Df(2L)Exel6038	Df(2R)ED1770	Df(3L)ED230	Df(3R)ED2
Df(2L)BSC188	Df(2L)Exel6277	Df(2R)ED1791	Df(3L)ED4196	Df(3R)ED50003
Df(2L)BSC204	Df(2L)Exel7011	Df(2R)ED2219	Df(3L)ED4287	Df(3R)ED5100
Df(2L)BSC208	Df(2L)Exel7034	Df(2R)ED2247	Df(3L)ED4293	Df(3R)ED5147
Df(2L)BSC209	Df(2L)Exel7070	Df(2R)ED2354	Df(3L)ED4341	Df(3R)ED5156
Df(2L)BSC213	Df(2L)Exel8038	Df(2R)ED2426	Df(3L)ED4421	Df(3R)ED5177
Df(2L)BSC214	Df(2L)J39	Df(2R)ED2457	Df(3L)ED4457	Df(3R)ED5330
Df(2L)BSC227	Df(2L)lt109	Df(2R)ED2487	Df(3L)ED4470	Df(3R)ED5339
Df(2L)BSC233	Df(2L)M24F-B	Df(2R)ED2747	Df(3L)ED4475	Df(3R)ED5428
Df(2L)BSC240	Df(2L)r10	Df(2R)ED3385	Df(3L)ED4486	Df(3R)ED5474
Df(2L)BSC241	Df(2L)tkv3	Df(2R)ED3610	Df(3L)ED4502	Df(3R)ED5514
Df(2L)BSC244	Df(2R)14H10W-35	Df(2R)ED3683	Df(3L)ED4543	Df(3R)ED5577
Df(2L)BSC252	Df(2R)BSC132	Df(2R)ED3728	Df(3L)ED4674	Df(3R)ED5578
Df(2L)BSC256	Df(2R)BSC135	Df(2R)ED3791	Df(3L)ED4710	Df(3R)ED5623
Df(2L)BSC277	Df(2R)BSC152	Df(2R)ED50004	Df(3L)ED4858	Df(3R)ED5644
Df(2L)BSC278	Df(2R)BSC161	Df(2R)Exel6061	Df(3L)ED4978	Df(3R)ED5705
Df(2L)BSC291	Df(2R)BSC19	Df(2R)Exel6062	Df(3L)ED50002	Df(3R)ED5718
Df(2L)BSC292	Df(2R)BSC199	Df(2R)Exel6064	Df(3L)ED5017	Df(3R)ED5780
Df(2L)BSC295	Df(2R)BSC267	Df(2R)Exel6066	Df(3L)Exel6085	Df(3R)ED5815
Df(2L)BSC354	Df(2R)BSC273	Df(2R)Exel6069	Df(3L)Exel6109	Df(3R)ED5938
Df(2L)BSC37	Df(2R)BSC274	Df(2R)Exel6284	Df(3L)Exel6112	Df(3R)ED6025
Df(2L)BSC454	Df(2R)BSC280	Df(2R)Exel7130	Df(3L)Exel6132	Df(3R)ED6085
Df(2L)BSC455	Df(2R)BSC281	Df(2R)Exel7149	Df(3L)M21	Df(3R)ED6096
Df(2L)BSC50	Df(2R)BSC298	Df(2R)Exel7162	Df(3L)ZN47	Df(3R)ED6220

Df(2L)BSC6	Df(2R)BSC303	Df(2R)Exel8057	Df(3R)10-65	Df(3R)ED6232
Df(2L)BSC688	Df(2R)BSC305	Df(2R)Kr10	Df(3R)A113	Df(3R)ED6255
Df(2L)BSC689	Df(2R)BSC307	Df(2R)M41A10	Df(3R)Antp17	Df(3R)ED6280
Df(2L)BSC690	Df(2R)BSC308	Df(2R)M60E	Df(3R)BSC137	Df(3R)ED6346
Df(2L)BSC692	Df(2R)BSC331	Df(2R)X1	Df(3R)BSC140	Df(3R)ED6361
Df(2L)BSC781	Df(2R)BSC347	Df(2R)X58-12	Df(3R)BSC141	Df(3R)ED7665
Df(2L)BSC812	Df(2R)BSC355	Df(3L)1-16	Df(3R)BSC321	Df(3R)Exel6154
Df(2L)BSC892	Df(2R)BSC356	Df(3L)6B-29+Df(3R)6B-29	Df(3R)BSC43	Df(3R)Exel6155
Df(2L)C144	Df(2R)BSC361	Df(3L)AC1	Df(3R)BSC464	Df(3R)Exel6159
Df(2L)dpp[d14]	Df(2R)BSC383	Df(3L)Aprt-32	Df(3R)BSC469	Df(3R)Exel6196
Df(2L)drm-P2	Df(2R)BSC425	Df(3L)BSC113	Df(3R)BSC47	Df(3R)Exel6197
Df(2L)ed1	Df(2R)BSC427	Df(3L)BSC117	Df(3R)BSC476	Df(3R)Exel6201
Df(2L)ED105	Df(2R)BSC429	Df(3L)BSC119	Df(3R)BSC489	Df(3R)Exel6202
Df(2L)ED1102	Df(2R)BSC485	Df(3L)BSC181	Df(3R)BSC497	Df(3R)Exel6203
Df(2L)ED1203	Df(2R)BSC550	Df(3L)BSC220	Df(3R)BSC501	Df(3R)Exel6264
Df(2L)ED1272	Df(2R)BSC595	Df(3L)BSC224	Df(3R)BSC502	Df(3R)Exel6270
Df(2L)ED1315	Df(2R)BSC597	Df(3L)BSC27	Df(3R)BSC503	Df(3R)Exel6272
Df(2L)ED136	Df(2R)BSC598	Df(3L)BSC289	Df(3R)BSC504	Df(3R)Exel7328
Df(2L)ED1378	Df(2R)BSC599	Df(3L)BSC368	Df(3R)BSC507	Df(3R)Exel7378
Df(2L)ED1473	Df(2R)BSC604	Df(3L)BSC371	Df(3R)BSC515	Df(3R)FDD-031795
Df(2L)ED19	Df(2R)BSC608	Df(3L)BSC375	Df(3R)BSC517	Df(3R)P115
Df(2L)ED247	Df(2R)BSC630	Df(3L)BSC388	Df(3R)BSC547	Df(3R)R133
Df(2L)ED250	Df(2R)BSC651	Df(3L)BSC389	Df(3R)BSC549	Df(3R)Tpl10
Df(2L)ED3	Df(2R)BSC661	Df(3L)BSC391	Df(3R)BSC567	Df(3R)Ubx109
Df(2L)ED334	Df(2R)BSC664	Df(3L)BSC411	Df(3R)BSC619	Df(3R)X3F
Df(2L)ED385	Df(2R)BSC701	Df(3L)BSC414	Df(3R)BSC621	

Deficiencies are organised in alphabetical order, and ones that generated no, few or sick progeny when crossed to the stable heteroplasmic females are underlined.

Table S2. A deficiency screen identified 38 nuclear loci carrying genes that affect mtDNA competition. Related to Figure 1.

Deficiencies	Total tested	Sick lines	*mt:yak* ≤ 2%	*mt:yak* ≥ 10%
2nd chromosome	184	25	30	3
3rd chromosome	155	38	3	2
Total	339	63	33	5

Table S3. A list of deficiencies that reduced *mt:yak* to ≤ 2%, increased *mt:yak* to ≥ 10%. Related to Figure 1.

Deficiency	Percent of *mt:yak*	Deficiency	Percent of *mt:yak*
mt:yak ≤ 2%			
Df(2L)BSC107	1.3	Df(2R)BSC280	1.85±0.32
Df(2L)BSC110	1.67±0.38	Df(2R)BSC361	1.78±0.11
Df(2L)BSC111	1.86±0.82	Df(2R)BSC383	1.44±1.23
Df(2L)BSC169	1.59±0.14	Df(2R)BSC425	1.44±0.16
Df(2L)BSC204	1.61±0.52	Df(2R)BSC597	1.60±0.55
Df(2L)BSC256	1.62±0.35	Df(2R)BSC630	1.94±0.13
Df(2L)BSC455	1.71±0.51	Df(2R)BSC769	1.55±0.26
Df(2L)BSC689	2.01±0.01	Df(2R)BSC880	1.23±0.92
Df(2L)BSC692	2.02±0.01	Df(2R)ED1725	1.11
Df(2L)ED678	1.93±0.14	Df(2R)ED1791	1.56±0.39
Df(2L)Exel6005	1.99	Df(2R)Exel6061	1.92±0.11
Df(2L)Exel7070	1.35±0.51	Df(3R)Exel6159	1.82
Df(2R)BSC267	1.64	Df(3R)Exel6264	2.00
Df(2R)BSC273	2.11±0.91	Df(3R)Exel6272	1.36
mt:yak ≤ 1%			
Df(2L)BSC149	0.66±0.68	Df(2R)BSC651	0.91±0.31
Df(2L)BSC240	0.03±0.03	Df(2R)BSC699	0.34±0.18
Df(2L)BSC241	0.44±0.38		
mt:yak ≥ 10%			
Df(2L)BSC252	29.96±3.16	Df(3R)Tpl10	12.74
Df(2L)BSC812	26.25±2.46	Df(3L)BSC730	10.14
Df(2L)C144	12.91±3.93		

All the deficiencies were tested at least twice. For those tested by three independent experiments, the percentage of *mt:yak* is presented as mean ± SD.

Table S4. Primers used in this study. Related to STAR Methods.

Primers for qPCR		
Heteroplasmy line	Common primer set	Specific primer set
mt:yak*/mt:ND2*^{del1}+*CoI*^{T300I}	cttttaatggttaaattccatttata	aaatcatattgaacctaaaaataatg
	ttattattacaatgaaaatgtaaggt	attctttgaataaatctctattaaat
mt:ATP6[1]*/mt:ND2*^{del1}+*CoI*^{T300I}	aattttatcagttattattggagc	gtaaagaagtttgatttaatcc
	tcaattaatcatttagggtgaatatt	tgattctctaattattaaagatctt
mt:ND2^{del1}**/*mt:CoI*^{T300I}** and	aattttatcggttattattggagc	tcaattaatcatttaggatgaatatt
**wild-type/***mt:ND2*^{del1}	gtaaagaagtttgatttagtcc	tgattctctaattattaaagatctt

Primers for RT-qPCR		
Gene	Primer set	Elongation temperature
Act42A	caccatgaagattaagattgttg	53°C
	aacagagtacttgcgttc	
*EF1*α	gcgtgggtttgtgatcagtt	60°C
	gatcttctccttgcccatcc	
tam	tggaggacagggcctacag	60°C
	tcttccaggcgattgaacatg	
pol γ-β	aaccgactgtcataaggt	53°C
	gtgtaagagcagggattg	

The mitochondrial genotype recognised by the specific primer set in each heteroplasmic line is highlighted in bold.

Supplemental References

S1 Bratic A, Kauppila TES, Macao B, Groenke S, Siibak T, Stewart JB, et al. Complementation between polymerase- and exonuclease-deficient mitochondrial DNA polymerase mutants in genomically engineered flies. Nat Commun 2015;6. doi:10.1038/ncomms9808.

S2 Samstag CL, Hoekstra JG, Huang C-H, Chaisson MJ, Youle RJ, Kennedy SR, et al. Deleterious mitochondrial DNA point mutations are overrepresented in *Drosophila* expressing a proofreading-defective DNA polymerase gamma. PLoS Genet 2018;14. doi:10.1371/journal.pgen.1007805.

S3 Kaguni LS, DNA polymerase gamma, the mitochondrial replicase, Annual Review of Biochemistry 2004 (73):293-320
